# A decision analysis model for material selection using simple ranking process

**DOI:** 10.1038/s41598-023-35405-z

**Published:** 2023-05-27

**Authors:** Shervin Zakeri, Prasenjit Chatterjee, Dimitri Konstantas, Fatih Ecer

**Affiliations:** 1 Geneva School of Economics and Management, University of Geneva, 1211 Geneva, Switzerland; 2 Faculty of Civil Engineering, Institute of Sustainable Construction, Laboratory of Operational Research, Vilnius Gediminas Technical University, Vilnius, Lithuania; 3 Sub-Department of Operations Research, Faculty of Economics and Administrative Sciences, Afyon Kocatepe University, Afyonkarahisar, Turkey

**Keywords:** Engineering, Materials science, Mathematics and computing

## Abstract

A large number of materials and various criteria fashion material selection problems as complex multi-criteria decision-making (MCDM) problems. This paper proposes a new decision-making method called the simple ranking process (SRP) to solve complex material selection problems. The accuracy of the criteria weights has a direct impact on the outcomes of the new method. In contrast to current MCDM methods, the normalization step has been eliminated from the SRP method as a potential source of producing incorrect results. The application of the method is appropriate for situations with high levels of complexity in material selection because it only considers the ranks of alternatives in each criterion. The first scenario of vital-immaterial mediocre method (VIMM) is used as a tool to derive criteria weights based on expert assessment. The result of SRP is compared with a number of MCDM methods. In order to evaluate the findings of analytical comparison, a novel statistical measure known as compromise decision index (CDI) is proposed in this paper. CDI revealed that the MCDM methods’ outputs for solving the material selection could not be theoretically proven and requires to be evaluated through practice. As a result, the dependency analysis-an additional innovative statistical measure is introduced to demonstrate the reliability of MCDM methods by assessing its dependency on criteria weights. The findings demonstrated that SRP is extremely reliant on criteria weights and its reliability rises with the number of criteria, making it a perfect tool for solving challenging MCDM problems.

## Introduction

Material selection problems and multi-criteria decision-making (MCDM) methods have strong relationships. Selecting suitable materials is the most challenging task in designing and developing new products^1^. Engineering design revolves around the objectives of achieving high performance, minimizing costs, and being environmentally conscious, which are often constrained by materials. Therefore, one of the key goals of optimal product design is the selection of materials that fulfill the design criteria while delivering the highest level of performance at the most economical cost^2,3^. Material selection is a complex decision-making process that involves the selection of the most suitable materials from a range of available alternatives based on multiple criteria. Multi-Criteria Decision Making (MCDM) refers to a class of mathematical methods used to solve such complex decision-making problems. These problems often arise in real-world situations where decision-makers has to select from a set of alternatives that differ across several criteria or dimensions. The goal of MCDM is to identify the best possible alternative or set of alternatives based on the decision-makers' preferences and priorities. MCDM problems are typically represented in a matrix format, where each row represents an alternative and each column represents a criterion. The elements of the matrix correspond to the performance of each alternative on each criterion. The decision-makers are then asked to provide weights or priorities for each criterion, indicating the relative importance of each criterion in the decision-making process. Material selection problems also involve the evaluation and selection of the most suitable material from a set of alternatives based on multiple criteria. This type of problem requires a decision-maker to weigh the importance of each criterion and to evaluate the alternatives accordingly. Material selection decisions typically involve multiple conflicting criteria, such as cost, performance, durability, physical and engineering properties, environmental factors, cost, and manufacturability, among others. These criteria may have different units of measurement, making it challenging to compare and evaluate alternatives using a single metric. Since the material selection could be converted into an MCDM problem-form problem, MCDM methods are suitable solutions to find the best material to meet the needs of the design and development of products. Many researchers have drawn attention to the connection between MCDM methods and material selection problems. According to^4^, among various methods and techniques that are employed to select the most suitable material for different projects, MCDM methodologies are among the most popular approaches. Reference^5^ call material selection problems MCDM dilemmas, and^6^ believe that MCDM methods are the only solution for the material selection problems that incorporate a large number of competing performance characteristics and are involved with many decision-makers. Similar to the latter authors^7^, argues that MCDM methods are efficient tools for effectively managing material selection problems incorporating various material properties and varied criteria. Reference^8^ also mentioned that MCDM methods aid in achieving the desired results from a product since the methods evaluate the materials’ performance under conflicting criteria. The use of MCDM methods in material selection problems has several advantages. First, it provides a systematic and objective approach to evaluate and rank materials based on multiple criteria. Second, it helps decision-makers to identify the critical criteria that have the most significant impact (having highest weight value) on the decision. Third, it enables decision-makers to evaluate and compare the performance of different materials under different scenarios. Finally, it provides a transparent and structured approach to the decision-making process, which can help to build consensus and improve communication among stakeholders. MCDM methods are widely used in material selection problems for computing the criteria weights and determining the rank of materials. Different scholars have proposed various categories for these two tasks. MCDM weighting methods can generally be classified into two categories: subjective weighting methods and objective weighting methods. The subjective weighting methods rely solely on human opinions, expectations, and judgments to assign weights to the criteria, whereas the objective weighting methods extract the criteria weights from the matrix of the decision-making problem. The ranking methods are divided into four subcategories, including the outranking methods, compromise ranking methods, distance-based methods, and the methods that use pairwise comparison. The complexity of an MCDM problem is associated with:

1. The complexity of input, involving objective and subjective values,
2. Different numbers of goals involved in the evaluation process of the alternatives,
3. Confliction between the nature of the criteria in the MCDM problems with multiple layers of criteria,
4. The number of non-beneficial criteria,
5. The number of criteria.

In this case, material selection problems always involve many alternatives and criteria, where with increasing the number of criteria, the reliability of the MCDM methods used for ranking the material decreases. To solve this problem, this paper proposes new MCDM method, called simple ranking process (SRP) which is based on ranking the alternatives against each criterion. The precision of criteria weight estimation directly affects the effectiveness of SRP algorithm. The new method is designed to deal with complex decision-making problems using simple processes compared to the existing MCDM methods. In this paper, the new method is applied to solve a material selection problem. Criteria weights of the problem are reassessed by the seven experts through a group decision-making process, followed by the application of vital-immaterial mediocre method (VIMM) to provide accurate weights. VIMM, which was proposed by^9^, is a subjective weighting method that was developed to bridge the structural and processual gaps in AHP and BWM. The paper presents several contributions centered around a new MCDM method that can effectively address complex decision-making problems. Reliability of the method is also shown to increase as the complexity of the problem increases. Additionally, the paper introduces two new statistical measures for validating results of MCDM methods. The paper is structured as follows: the second section presents the literature review, while the third section describes the proposed method and VIMM. The fourth section applies the methods to a real-world material selection problem. The fifth section discusses the results and introduces a new statistical measure to validate complex MCDM solutions. The paper concludes in the sixth section with a summary of the findings and suggestions for future research.

## Literature review

Dissimilar mathematical treatments are employed by different MCDM methods based on the categories they are members of to derive the best material, consequently offering different materials as the most suitable option for the same problem. With an extensive literature review, this section aims to provide a scientific perspective for the readers regarding the MCDM methods application in material selection problems and the gaps in the current course of MCDM methods and material selection engagement. Specifically, this section seeks to demonstrate the following:

1. The investigation of the relationship between MCDM methods in the different categories with the different complex material selection problems,
2. The prevalent problem of dissimilarities between the outputs of the MCDM methods in solving material selection problems that are revealed by different studies, which emerges as the results validation issues,
3. Solutions the studies employed to overcome the dissimilarities.

**Figure 1:**
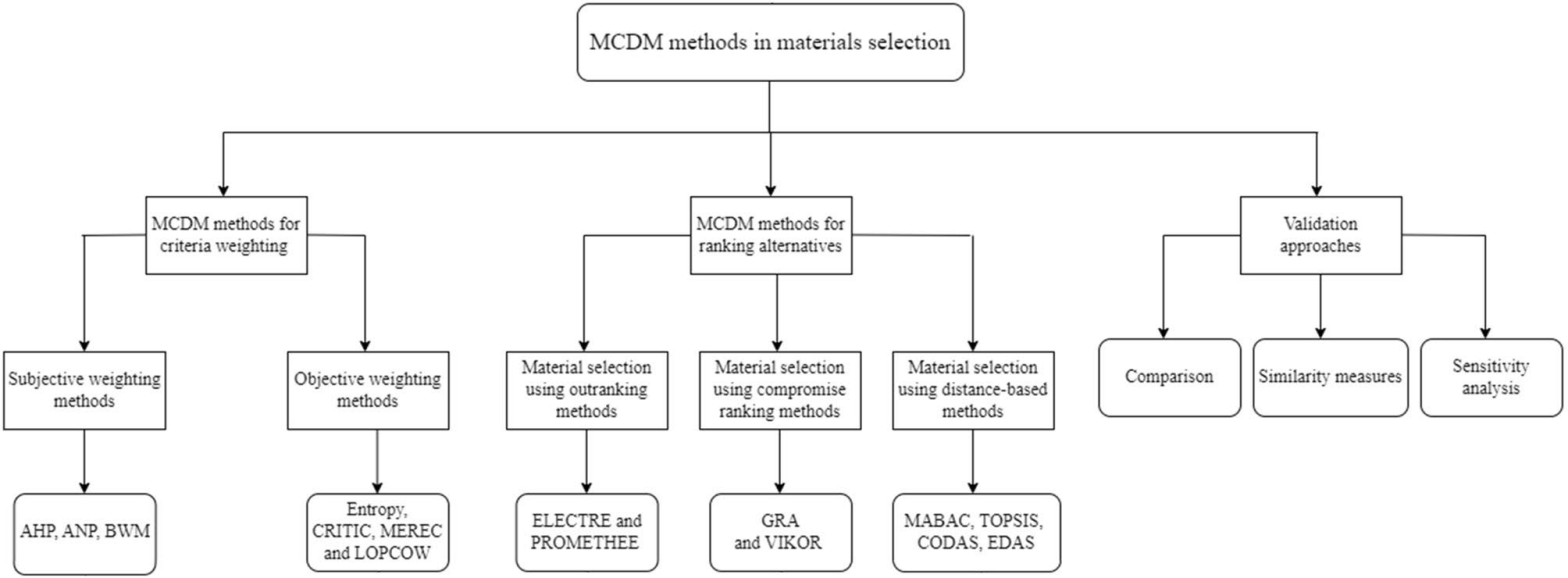
The literature review structure.

These gaps will be later addressed in the paper. To visualize the literature review section's composition, its structure has been illustrated in Fig. 1.

### MCDM methods for material selection problems

Appropriate material selection results in improved quality and enhanced product life cycle, while inaccurate selection leads to increased design cost, lack of productivity, poor end product performance, critical component damage, and eventually untimed product failure^10^. Thus, it is critical to exert a method that optimizes material selection decisions and minimizes the risk of poor selection. This paper proposes a new MCDM method to solve this problem. So far, several MCDM methods for material selection problems have been developed and applied. The technique for order of preference by similarity to ideal solution (TOPSIS) developed by Hwang and Yoon^11^, analytic hierarchy process (AHP) by Saaty^12^, analytical network process (ANP) proposed by Saaty^13^^,^ simple additive weighted (SAW) (MacCrimmon & Rand.^14^^,^ data envelopment analysis (DEA) proposed by Charnes^15^, Vise Kriterijumska Optimizacija I Kompromisno Resenje (VIKOR) developed by Opricovic and Tzeng^16^, decision making trial and evaluation laboratory (DEMATEL) developed by Fontela and Gabus^17^, preference ranking organization method for enrichment evaluations (PROMETHEE)^18^ and ELimination Et Choix Traduisant la REalité or ELimination and Choice expressing reality (ELECTRE) proposed and advocated by Roy^19^ are some of the most popular MCDM methods for material selection problems. Some of the recent developments in MCDM methods are ranking based on optimal points multi-criteria decision-making method (RBOP) by Zakeri.^20^^,^ step-wise weight assessment ratio analysis (SWARA) first developed by Keršulienė & Turskis^21^, subjective weighting method using continuous interval scale by Toloie-Eshlaghy et al*.*^22^^,^ superiority and inferiority ranking (SIR) method by Xu^23^, multi-attribute evaluation using imprecise weight estimates (IMP) method proposed by Jessop^24^, and best–worst method (BWM) introduced by Rezaei^25^.

In some problems, the outputs of MCDM methods are dissimilar^26,27^. The comparison of MCDM methods and their outputs can be found in Refs.^28,29^. To evaluate alternative hydropower systems on the “Drina River,” Opricovic & Tzeng^28^ compared the extended VIKOR method with TOPSIS, PROMETHEE, and ELECTRE, where the TOPSIS and VIKOR generated the same ranking for the two best alternatives while the ratio was different. By utilizing Kendall’s tau-b test and Spearman’s rho test to determine the significance of rank correlation between the compared methods, the sensitivity of final ranks to selected fuzziness intervals, and the sensitivity of similarities and dissimilarities of different decision ranking methods to the dimensions of the decision matrix, the appropriate MCDM method was selected in the work of Zamani-Sabzi et al.^29^. They evaluated the performances of ten popular MCDM methods including SAW, weighted product method (WPM), compromise programming (CP), TOPSIS, AHP, VIKOR, and ELECTRE under a fuzzy environment. According to Ref.^30^, the inconsistency that occurred in generating dissimilar results by MCDM methods is due to four reasons:

1. The methods use weights differently in their calculations.
2. Algorithms differ in their approach to selecting the ‘best’ solution.
3. Many algorithms attempt to scale the objectives, which affects the weights already chosen.
4. Some algorithms introduce additional parameters that affect the selection of the solution.

The most important reasons that directly affect the final output of the MCDM methods are 1. Determining criteria weights; and 2. Policies/philosophies for evaluating alternatives. The application of these two items to material selection problems has been discussed in the following sections.

#### MCDM methods for criteria weighting

One of the main challenges in MCDM problems is to determine the relative importance of each criterion. Criteria weighting methods are used to assign weights to the criteria, reflecting their relative importance in the decision-making process. Criteria weighting methods in MCDM environment are mostly divided into subjective and objective methods. There is also a third type of weighting method, popularly known as combinative weighting method, which utilizes hybridization or integration of different subjective and objective methods using multiplication and additive synthesis^31^. Subjective weighting methods depend on DMs’ judgments, levels of knowledge, perception, and intentions,and these methods do not use a formal mathematical approach to determine the weights, but rather rely on the experience and expertise of the decision-makers. Subjective weighting methods are often used when there is a lack of data or when the criteria are difficult to quantify. On the other hand, objective weighting methods extract weights directly from decision matrix using mathematical algorithms without considering human judgments to avoid inaccuracies and imprecisions. In order to reduce errors and ambiguities in the decision-making process, hybrid weighting methods (subjective and objective methods) have also been developed. Some of the most popular subjective methods are Digital Logic and Modified Digital Logic methods^32^, Pairwise Comparison (e.g. AHP), Best–worst Method, Ratio method^33^_,_ Swing method^34^, Simple multi-attribute ranking technique (SMART) and SIMOS method^35^. Some of the most popular objective weighting methods are Shannon’s entropy^36^ and CRITIC (The CRiteria Importance Through Intercriteria Correlation) methods^37^. An overview of MCDM weighting methods for material selection problems has been presented briefly in the following sections.

##### Subjective weighting methods in materials selection

Some examples of the subjective weighting methods in the complex material selection problems to evaluate the importance of the selected criteria are shown in Table 1. AHP and BWM methods are observed to be very popular methods for criteria weighting in material selection problems. AHP provides a systematic and structured approach to decision-making and helps to organize complex decision problems in a hierarchical structure, making it easier for decision-makers to understand the problem and the relationships between the criteria. On the other hand, BWM is a straightforward method that requires decision-makers to select the best and worst criteria from a set of options, making it easy to understand and apply.

**Table 1:** Subjective weighting methods and their applications in materials selection.

Author(s)	Case application	Criteria	Subjective weighting method
Das et al.^38^	Material selection case study of spur gear reduction unit	1. The pressure angle, 2. Module, 3. Number of teeth to avoid interference, 4. Gear width, and 5. Gear material	AHP
Mahmoudkelaye et al.^39^	To select sustainable materials for building enclosures	Economic, technical, environmental, and socio-cultural and their corresponding sub-criteria	ANP
Patnaik et al.^10^	To select the best composite materials for wear-resistant applications	1. Physical properties, 2. Mechanical properties, 3. Slurry abrasion, 4. Wear properties	AHP
Prasad et al.^40^	Coating material for magnesium alloy	1. Density, 2. Thermal conductivity, 3. Thermal expansion coefficient hardness, 4. Young’s modulus elastic recovery, 5. Critical load, 6. Yield stress, 7. Melting temperature, 8. H/E ratio H3/E2 ratio, 10. Wear resistance, 11. Coefficient of friction, 12. Radiation sensitivity, 13. Workability, 14. Appearance, 15. Oxidation resistance, 16. Oxidation rate constant, 17. Impact resistance, 18. The possibility of surface treatment material, 19. Manufacturing, 20. Availability, 13. Accessibility, 14. Toxicity, 15. Adhesion to the substrate, 16. Bond strength, 17. Durability, 18. Brittleness, 19. Compatibility of the material, 20. Matrix, 21. Framed, 22. Mixed, 23. The aging tendency, 24. Porosity, 25. Geographical location, 26/political stability & foreign policy, 27. Exchange rate & economic position	Fuzzy AHP
Palanisamy et al.^41^	Additive manufacturing machine and materials	1. Cost, 2. Visual and aesthetic modeling, 3. Tensile strength, 4. Shore hardness, 5. Mixing number, 6. Number of digital materials, 7. Frequent order, and 8. Elongation at break	BWM
Maghsoodi et al.^42^	Phase change material selection for interior building surface application	1. Melting temperature, 2. Latent heat storage capacity, 3. Thermal conductivity, 4. Specific heat capacity, 5. Energy density and 6. Cost	BWM
Yang et al.^43^	Phase change material selection for solar domestic hot water system	1. Latent heat, 2. Density, 3. Specific heat for solid, 4. Specific heat for liquid, 5. Thermal conductivity and 6. Cost	AHP
Kumar et al.^44^	Coating material selection in tooling industries	1. Indentation hardness, 2. Young’s modulus, 3. Wear resistance, 4. Plastic Deformation, 5. Strain hardening exponent, 6. Coefficient of thermal expansion, 7. Surface roughness, 8. Coefficient of friction 9. Wear rate	BWM
Aksakal et al.^4^	Thermal insulation material selection	1. Thermal conductivity, 2. Periodic thermal transmittance, 3. Specific heat, 4. Density, 5. Decrement factor, 6. Surface mass, 7. Thermal transmittance, 8. Thermal wave shift	Fuzzy BWM
Grachev et al.^45^	Dental material selection in manufacturing removable dentures	1. Mechanical properties, 2. Biological properties, 3. Tribological properties, 4. Technological properties and 5. Cost	AHP

##### Objective weighting methods in materials selection

Shannon's entropy method is one of the most popular objective methods for computing criteria weights for material selection applications. Compared to other objective weighting methods, most studies employed Shannon’s entropy to compute the criteria weights in this category. Apart from Entropy method, there are other recently developed objective weighting methods like method based on the removal effects of criteria (MEREC) and logarithmic percentage change driven objective weighting (LOPCOW). Table 2 shows instances of using objective weighting methods in material selection problems.

**Table 2:** Objective weighting methods and their applications in materials selection.

Author(s)	Case application	Criteria	Objective weighting method
Bhowmik et al.^46^	Energy-efficient materials	1. Density, 2. Bulk Modulus, 3. Compressive Strength, 4. Thermal Conductivity, 5. Thermal Expansion, 6. Resistivity, 7. Cost, 8. Energy Production, and 9. CO_2_ Emission	Entropy
Oluah et al.^47^	Latent heat storage materials for optimal performance of a Trombe wall	1. Heat of Fusion, 2. Thermal Conductivity, 3. Density, and 4. Cost	Entropy
Aksakal et al.^4^	Thermal insulation material	1. Thermal Conductivity, 2. Periodic Thermal Transmittance, 3. Specific Heat, 4. Density, 5. Decrement Factor, 6. Surface Mass, 7. Thermal Transmittance, and 8. Thermal Wave Shift	CRITIC
Mahajan et al.^48^	Natural Fiber for Sustainable Composite	1. Aspect Ratio, 2. Strain at break, 3. Specific strength, 4. Specific modulus, 5. Moisture Absorption, and 6. Cost	Entropy and CRITIC
Akgün et al.^49^	Selection of most appropriate carbon-based nanomaterials	1. Melting Point Temperature Change, 2. Latent Heat Change, 3. Thermal Conductivity Enhancement, 4. Leakage, 5. Greenhouse Gas, 6. Cost, and 7. Agglomeration	Entropy
Haq et al.^50^	Material selection for wing-spar of human-powered aircraft	1. Price, 2. Tensile Strength, 3. Young’s Modulus, 4. Density, 5. Compressive Strength, 6. Creep Resistance, 7. Fatigue Resistance, 8. Machinability, 9. Recyclability and 10. Carbon Footprint During Manufacture	Entropy
Ulutaş et al.^51^	Building insulation material selection	1. vapour diffusion resistance factor, 2. sound absorption coefficient, 3. embodied carbon, 4. embodied energy, 5. cost, 6. reaction to fire, 7. specific heat capacity, 8. thermal conductivity, and 9. density	MEREC and LOPCOW

#### MCDM methods for ranking alternatives

MCDM methods are generally developed for ranking alternatives based on four main concepts:

1. Outranking methods such as ELECTRE, PROMETHEE, and GLDS (Gain and Lost Dominance Score).
2. Compromise ranking policies such as VIKOR, GRA.
3. Distance-based methods such as TOPSIS, Evaluation based on Distance from Average Solution (EDAS), Multi-Attributive Border Approximation Area Comparison (MABAC) and Combinative Distance-based Assessment Method (CODAS).
4. Pairwise comparison such as AHP and ANP.

There are other categorizations suggested by scholars, e.g. the classification of the MCDM methods into five classes^52^^,^ including the quantitative methods, qualitative methods, mixed techniques, heuristics, and metaheuristics, and simulation,or categorizing them into the three main groups^53^ including the utility value-based methods, the outranking methods, preference ordering based methods. Except for the pairwise comparison methods, which have been discussed earlier, in the following sections, the application of MCDM methods to material selection problems has been briefly reviewed based on the three MCDM categories comprising outranking, compromise ranking, and distance-based ranking methods.

##### Material selection using outranking methods

Different forms of ELECTRE method have been applied to material selection problems. Using a hybrid method and the opinions of four experts in a group decision-making framework^54^, employed ELECTRE III to solve the office flooring selection problem, in which the ease of cleaning and maintenance, durability, quietness, style and comfort, sustainability, and cost-effectiveness have been used as criteria. Singh et al.^55^ employed a hybrid model of ELECTRE II and entropy to solve the friction composite selection problem consisting of seven attributes and nine alternatives. ELECTRE and PROMETHEE methods have also been used to solve material selection problems. Gul et al.^56^ proposed a solution for an automotive instrument panel material selection problem using fuzzy PROMETHEE method while considering Styrene Maleic Anhydride, Polycarbonate, Polypropylene, and Acrylonitrile Butadiene Styrene as the potential material alternatives, and maximum temperature limit, recyclability, elongation, weight, thermal conductivity, tensile strength, cost, and toxicity level as the criteria. Using six criteria containing creep strength, resistance to oxidation, thermal expansion coefficient, yield strength, limit strain, and toughness against five alternatives, Zindani & Kumar^57^ designed a PROMETHEE-GAIA method to find the best material suitable for the labyrinth seal strips. Exconde et al.^58^ focused on the selection of materials for use in 3D printer filaments using ELECTRE method. Singh et al.^55^ employed a hybrid ELECTRE II-entropy model for selecting natural fibers for use in brake friction composites. The results revealed that 10 wt% banana fiber emerged as the best alternative among all fibers that were considered. Mahajan et al.^48^ investigated the selection process of natural fibers for sustainable composites. They proposed a hybrid MCDM model that utilized the Entropy and CRITIC methods to calculate the criteria weights, and the PROMETHEE II, TOPSIS, and VIKOR methods to rank the materials under consideration. Bhaskar and Khan^1^ evaluated seven materials based on ten material selection criteria to identify the best polymer-based biomaterial for dental applications using ELECTRE, PROMTHEE, VIKOR, TOPSIS, and MOORA methods. Ranjith and Vimalkumar (2022) developed a hybrid MCDM method that integrated ELECTRE and MOORA methods for selecting the best electrode material from five available alternatives for electrical discharge machining of magnesium composites. Kirişci et al.^59^ extended the ELECTRE I model to the Fermatean fuzzy ELECTRE I model for group decision-making using Fermatean fuzzy human assessments for material selection of the femoral component of the hip joint prosthesis. Zhou^60^ adopted ELECTRE method to select an optimal recycled material for 3D printer filament from a waste plastic stream containing a mixture of polymers. The results indicated that recycled polyethylene terephthalate outperformed all other considered plastic materials.

##### Material selection using compromise ranking methods

The literature suggests that GRA and VIKOR are the most widely used compromise ranking methods for material selection applications. Jayakrishna and Vinodh^61^ proposed an integrated approach that employed GRA to rank materials based on cost, material properties, and environmental impact. Zhang et al.^62^ used GRA in combination with other methods, such as DEMATEL, ANP, and TOPSIS, to select the optimal green material for sustainable rubbish bins based on multiple criteria. Sanghvi et al.^63^ adopted a combined framework of GRA and fuzzy logic to leverage the benefits of both methods for bone staples material selection problem. Similarly, Wang & Li^64^ employed GRA in conjunction with a hybrid weighting method that integrated AHP, Fuzzy AHP, and quality function deployment (QFD) methods to solve the lightweight automotive body material selection problem. Dwivedi & Sharma^65^ applied an integrated entropy-CoCoSo method to identify the most suitable sustainable material for an engineering application. Maidin et al.^66^ developed a systematic evaluation framework using GRA to rank natural fiber materials as reinforcement composites for cyclist helmets, with pineapple identified as the most suitable candidate for optimal safety. In another study, Maidin et al.^67^ applied 6 Sigma and GRA methods to select the most suitable thermoplastic matrix for natural fiber composites in cyclist helmets, integrating qualitative and quantitative approaches to identify thermoplastic polyethylene as the ideal matrix.

Ishak et al.^68^ applied fuzzy VIKOR method to select the optimal natural fiber type for fiber-reinforced composites to be utilized in the manufacture of a fiber-metal laminate for car front hoods. The objective of this analysis was to achieve a reduction in transportation weight, which is crucial for improving fuel efficiency and reducing environmental impact. Dev et al.^69^ utilized an Entropy-VIKOR model to determine the optimal composite material for an automobile piston application case study. Entropy method was applied to compute the relative weights of various evaluation criteria, while VIKOR method was employed to rank the composite materials under consideration. The approach ensured a comprehensive and objective evaluation of the available alternative materials, leading to a well-informed decision regarding the most suitable composite material for the intended application. Gadhave et al.^70^ conducted a study on the selection of phase change material using three MCDM methods and utilized AHP—entropy methods to determine the criteria weights. VIKOR, TOPSIS and EXPROM2 methods were used to rank the alternative materials. The ranking was based on the compromised weight obtained through AHP and entropy methods. Bhaskar & Khan^1^ showcased the effectiveness of five different hybrid MCDM methods in identifying the optimal polymer-based biomaterial for use in dentistry. They evaluated seven materials based on ten criteria and utilized AHP to determine criteria weights. The materials were ranked using AHP-VIKOR, AHP-TOPSIS, AHP-MOORA, AHP-ELECTRE, and AHP-PROMTHEE methods. Grachev et al.^45^ developed a formalized method for selecting dental materials in the production of removable dentures. Their approach combined AHP-Extended VIKOR method and involved analyzing interval quantitative estimations. Bhuiyan & Hammad^71^ developed a decision support system to assist with selecting the most sustainable structural material using a hybrid MCDM method that combined AHP, TOPSIS, and VIKOR in a fuzzy environment.

##### Material selection using distance-based methods

Applications of distance-based MCDM methods are well popular in solving material selection problems. Xue et al.^72^ introduced a new method based on interval-valued intuitionistic fuzzy sets and MABAC to address the issue of incomplete weight information in automotive instrument panel material selection problems. Tian et al.^73^ proposed a hybrid MCDM approach that combined AHP and grey correlation TOPSIS (GC-TOPSIS) methods to select the optimal green decoration materials from a pool of 10 different types of solid woods. Ahmed et al.^74^ proposed a decision support framework taking into account technical, environmental, social, and economic criteria for ranking concrete materials. The framework comprised of an optimal scoring method (OSM) that shortlisted the materials, followed determination of ranking orders using AHP-TOPSIS method. Deshmukh & Angira.^75^, used VIKOR and TOPSIS to solve the material selection problem for radio-frequency microelectromechanical system (RF-MEMS) shunt capacitive switches considering Young’s Modulus, Electrical resistivity, Thermal conductivity, Fracture strength as the criteria. Maghsoodi et al.^76^ addressed a material selection problem in the context of dam construction projects by proposing a hybrid decision-making approach that combined SWARA and CODAS methods. The proposed approach considered target-based attributes to facilitate the evaluation process. Roy et al.^77^ developed an evaluation framework for solving sustainable material selection problems in construction projects with incomplete weight information. The approach extended CODAS method by incorporating interval-valued intuitionistic fuzzy numbers. Yadav et al.^78^ proposed a novel MCDM approach based on TOPSIS-PSI for choosing the best alternative material in marine conditions. Dhanalakshmi et al.^79^ employed a comprehensive MCDM-based approach, combining Fuzzy AHP, TOPSIS, and EDAS methods, for the selection of pyrolysis materials. The criteria were defined based on the objective of achieving maximum bio-oil yield during pyrolysis. Kar & Jha^80^ proposed a novel approach that integrates material management with construction schedule to prioritize construction materials using ANP-TOPSIS method. The criteria weights were determined using ANP, while TOPSIS was used to calculate material criticality of the alternative materials. The result of the study showed that structural steel was the best material. Another hybrid method of VIKOR and TOPSIS could be found in Ref.^81^, where it is used to select the best dielectric material for RF-MEMS switches with low power consumption. Yang et al.^82^ developed a method for selecting an appropriate normalization method in TOPSIS when choosing the optimal tribological coating material. They also introduced entropy-based and variation coefficient-based performance scores to evaluate the effectiveness of the normalization method. Kumar et al.^83^ applied TOPSIS method to optimize the selection of glazing materials for solar thermal applications with multiple response characteristics. The study considered seven alternative materials and six criteria for material selection in the optimal design. The results showed that Polysulfone material was the best choice for solar thermal applications. Aires & Ferreira^84^ developed a decision-making framework for selecting the most suitable thermal insulation material for enhancing energy efficiency, by integrating Fuzzy BWM, CRITIC, and Mixed Aggregation by Comprehensive Normalization Technique (MACONT). Among the considered alternatives, Polyisocyanurate was identified as the optimal material based on the defined criteria. Abishini & Karthikeyan^85^ investigated the use of AHP, TOPSIS, EDAS, VIKOR, and Taguchi-based super ranking concepts to select the optimal aluminum alloy material for the sheet metal forming process. The study revealed that AA2024 aluminum was the best-ranked material among the alternatives considered. Kazemian et al.^86^ conducted a comprehensive evaluation of ten different materials used for intraoral stents in head and neck cancer patients. The study aimed to identify the most suitable material using the TOPSIS method, and the results showed that Ethylene Vinyl Acetate was the best material. Sharma et al*.*^87^ investigated the optimal material selection problem for railway wagons using VIKOR, TOPSIS, PROMETTHEE, and WASPAS methods and also compared the relative performances. The study revealed aluminum alloy Al 6005-T6 as the most suitable material. Remadi & Frikha^88^ proposed a model for ranking green materials using CODAS method and utilized intuitionistic fuzzy sets within an uncertain group decision-making environment. Wankhede et al.^89^ focused on selection of natural fiber for long lasting composites using CODAS method. Basalt was found as the best natural for long lasting composites followed by flax and Kenaf respectively. Ranking performance of CODAS was also compared with MOORA method. Table 3 summarizes the distance-based studies which used the aforementioned methods. Based on the literature review as presented above, a number of MCDM methods have been employed to solve material selection problems. This leads us to the next part of the literature review, which focuses on the differences between the results of different MCDM methods. This section poses the question: "Which MCDM method is most suitable for solving complex material selection problems with a large number of alternatives and criteria?".

**Table 3:** Distance-based MCDM methods in materials selection.

Author	Case application	Distance-based MCDM method
Xue et al.^72^	Automotive instrument panel	Interval-valued intuitionistic fuzzy MABAC
Tian et al.^73^	Building decoration material selection (Solid wood)	Grey correlation-TOPSIS
Ahmed et al.^74^	Ranking concrete supplementary material	TOPSIS
Deshmukh & Angira^75^	Material selection problem for the bridge of RF-MEMS shunt capacitive switches	TOPSIS
Maghsoodi et al.^76^	Dam construction material selection	CODAS
Roy et al.^77^	Sustainable material selection in construction projects	Interval-valued intuitionistic fuzzy CODAS
Yadav et al.^78^	Material selection in marine applications	TOPSIS-PSI
Zhang et al.^90^	Bone transplant replacement material selection	TOPSIS
Dhanalakshmi et al.^79^	Pyrolysis material selection	TOPSIS and EDAS
Kar & Jha^80^	Construction material selection	TOPSIS
Yang et al.^82^	Tribological coating material selection	TOPSIS
Kumar et al.^83^	Glazing material for solar thermal application	TOPSIS
Aires & Ferreira^84^	Flywheel material selection	R-TOPSIS
Abishini & Karthikeyan^85^	Aluminum alloy material selection for sheet metal forming process	EDAS
Kazemian et al.^86^	Material selection of intraoral stents	TOPSIS
Sharma et al.^87^	Lightweight material for railway vehicles	TOPSIS
Remadi & Frikha^88^	Green material selection	Intuitionistic fuzzy CODAS
Wankhede et al.^89^	Selection of natural fiber for long lasting composites	CODAS

### Dissimilarities in ranking results for material selection problems

This section discusses the application of various MCDM methods to the same material selection problems, which reveal that decision-makers may have different rankings using different methods. For instance, Singh et al.^55^ used ELECTRE II to select the best material and compared the rankings derived using COPRAS, TOPSIS, VIKOR, SAW, MOORA, and PSI to validate the outputs. While all the methods identified a similar alternative as the best, the rankings were slightly different. Similarly, Hafezalkotob & Hafezalkotob^91^ compared the rankings produced by Target-based MULTIMOORA method with two different modes and their aggregate ranking of with other methods including Target-based TOPSIS, Target-based VIKOR and Interval target-based VIKOR methods. Despite generating different rankings, all methods, except for Target-based MULTIMOORA with an integrated significant coefficient (Mode 2), suggested the same material for the considered problem. However, the aggregate ranking methodology proposed two materials as the best. To analyze the similarity of MCDM outputs, Spearman rank correlation coefficient was also used which revealed differences in the obtained rankings. Mousavi-Nasab & Sotoudeh-Anvari^92^ presented five material selection examples and compared the rankings using DEA, VIKOR, TOPSIS, ELECTRE II, and COPRAS methods. In the first example, a significant difference in material ranking between DEA and other methods was observed, while in the second example, the results were more similar. Although the same material was selected by most methods, the rankings of the remaining materials varied significantly. The authors also concluded that for material selection problems, it is more reliable to use multiple MCDM methods instead of relying on a single technique. In example 3, differences were observed in the generated rankings and the selected materials. Similarly, the application of MCDM methods in the subsequent two examples demonstrated different ranking results and slight differences in the selected materials. The authors concluded that TOPSIS and COPRAS are more consistent, but using multiple MCDM methods to solve material selection problems is preferable. Another attempt to optimize decision-making in material selection problems can be found in the work of Zhang et al.^93^^,^ where they proposed a new MCDM method. It was evident that changes in methods for computing a component of the decision-making process, such as criteria weights, could significantly impact the final results. Fuzzy BWM and fuzzy G-VIKOR methods were employed by Zhang et al.^94^ to solve a material selection problem. To validate the results, a sensitivity analysis was conducted to explore the influence of criteria weights on the final ranking. In^54^ study, a sustainable building material selection problem was solved using ELECTRE III. The results obtained from ELECTRE III were compared with SAW, TOPSIS, COPRAS, and MULTIMOORA. Additionally, sensitivity analysis was performed to demonstrate the superior performance of ELECTRE III compared to other MCDM methods. Consequently, if other MCDM methods are utilized by DMs for any reason, a poor material selection result might have been obtained. Another significant difference between the rankings of materials generated by different MCDM methods is highlighted in the work conducted by Chatterjee et al.^95^. COPRAS and EVAMIX methods were employed for solving material selection problems, and the results obtained from these methods were compared with TOPSIS, VIKOR, and AHP in terms of calculation time, simplicity, transparency, possibility of graphical interpretation, and information type, instead of using sensitivity analysis or Spearman’s rank correlation coefficient. Through two examples, it was concluded that COPRAS and EVAMIX methods are applicable, capable, and accurate for solving material selection problems.

## Research methodology

**Figure 2:**
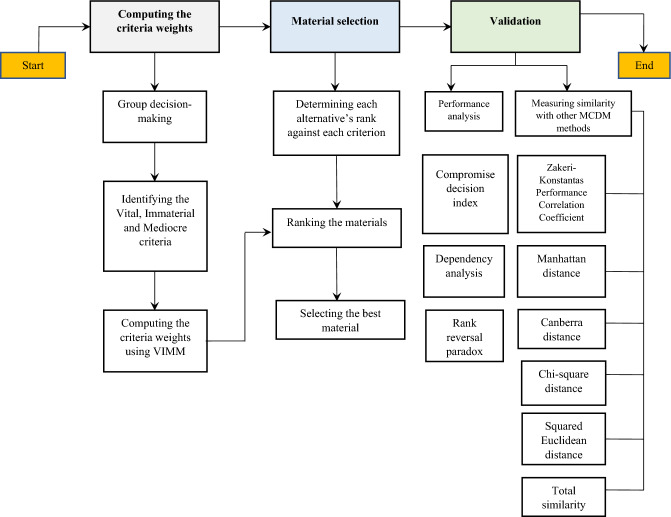
The methodological workflow.

The research methodology is constructed on three main procedures, 1. Computing the criteria weights; 2. Selecting the best material; and 3. Validating the obtained results. The methodological process and the tools used in this paper are shown in Fig. 2.

### Research tools

This section presents two main tools used to solve the material selection problem. It is divided into two sub-sections that address the issues mentioned previously. The first sub-section presents the new MCDM method called SRP. The second sub-section presents VIMM, which is a subjective weighting method used in the first scenario algorithm.

#### SRP

SRP method is a novel approach developed to solve MCDM problems. It is based on the ranks of alternatives in each criterion, which allows it to provide accurate and reliable outputs while avoiding the complexities of existing MCDM methods. Unlike other methods, SRP does not require a normalization process as it directly works with criteria weights. SRP has the following simple steps:

*Step 1* Defining criteria for the evaluation of the alternatives where ${A}_{i}$ signifies the alternatives, ${c}_{j}$ states the criteria, and $i=\left\{1,\dots ,m\right\}$ and $j=\left\{1,\dots ,n\right\}$.

*Step 2* Establishing the decision matrix, where ${X}_{ij}$ denote the decision matrix and ${r}_{ij}$ expresses the score of $i$th alternative against $j$th criterion.

1 $${X}_{ij}={r}_{ij}. $$

*Step 3* Determining the ranks of alternatives in each criterion where the ranking process is based on the higher value of ${r}_{ij}$ in the beneficial criteria ($\underset{1\le i\le m}{\mathrm{max}}{r}_{ij}$) and lower value of ${r}_{ij}$ in the non-beneficial criteria ($\underset{1\le i\le m}{\mathrm{min}}{r}_{ij}$). The following equation Eq. (2) demonstrates the new ranking matrix where ${X}_{ij}^{^{\prime}}$ is the ranking matrix and ${R}_{i}^{j}$ is the rank of $i$th alternative against $j$th criterion.

2 $${X}_{ij}^{^{\prime}}={R}_{i}^{j},$$

*Step 4* The fourth step is the construction of the weighted ranking matrix according to Eq. (3), where ${W}_{j}$ stands for the importance wrights of criteria and ${X}_{ij}^{"}$ shows the weighted ranking matrix.

3 $${X}_{ij}^{"}={{W}_{j}R}_{i}^{j},$$

*Step 5* Computing the total ranking score of the alternatives as follows:

4 $${SR}_{i}=\sum_{j=1}^{n}{{W}_{j}R}_{i}^{j},$$

*Step 6* Finally, prioritizing alternatives based on the higher value of ${\mathfrak{R}}_{i}$, where $m$ is the number of alternatives.

5 $${\mathfrak{R}}_{i}=m-{SR}_{i}.$$

#### VIMM: first scenario algorithm

VIMM is a subjective weighting method that is developed to use less pairwise comparison and also distance-based computations to extract the most accurate weights from the decision-makers' opinions and judgments. VIMM is designed based on three main elements called vital, immaterial, and mediocre criteria. The vital criterion plays the role of the most important criterion, which has the most impact in achieving the decision-making’s goal(s). It receives the highest value through computation. On the other hand, the immaterial criterion plays the opposite role and has the lowest impact on reaching the goal(s). The first vital and immaterial criteria select by the decision-maker(s), while the algorithm determines the following vital and immaterial criteria. There exists a third criterion, called the mediocre criterion. It refers to a criterion that affects reaching the decision-making goal to a lesser extent than the vital criteria and higher than the immaterial criterion. VIMM generates high-accuracy results and is more reliable than the popular MCDM weighting methods such as AHP.

The following phases are proposed by Zakeri et al*.*^9^ for the classic VIMM: the first scenario to calculate the weights of criteria in a one-goal decision-making problem.

*Step 1* The algorithm begins with determining the vital, immaterial, and mediocre criteria by the decision-makers.

*Step 2* After selecting the vital and immaterial criteria, the second step is to allocate five and one as the corresponding values for the vital and immaterial criteria, respectively.

**Figure 3:**
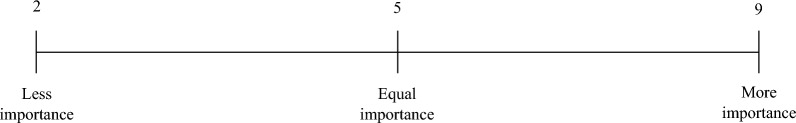
The linguistic/numeric variables scale.

*Step 3* Comparing the remaining criteria against the vital and immaterial criteria following the numerical-linguistic scale. To conduct the comparison, VIMM uses a scale to convert decision-makers’ subjective opinions into numbers within an interval of $\left[\mathrm{2,5},9\right]$, in which decision-maker is able to select any rational number between these three numbers. The scale is illustrated in Fig. 3.

*Step 4* Establishing the distance matrix by calculating the distances between each criterion and the vital and immaterial criteria according to the linguistic/numeric scale in (Fig. 2) is the fourth step of the VIMM algorithm. This step includes two steps:

*Step 4.1* Normalizing the distance matrix by following Eqs. (6), (7), where ${d}_{xy}^{+}$ and ${d}_{xy}^{-}$ stand for distances between the $y$th criterion in the $x$th comparison, and the immaterial and vital criteria respectively, $x$ states the number of comparison process, and $y$ stands for the number of criteria.

6 $${{d}_{xy}^{+}}^{^{\prime}}={c}_{y/\underset{y\in j}{\mathrm{max}}{d}_{xy}^{+}}, y\in j,$$

7 $${{d}_{xy}^{-}}^{^{\prime}}=\underset{y\in j}{\mathrm{min}}{d}_{xy/{d}_{xy}^{-}}^{-}, y\in j, $$

*Step 4.2* Computing the first score Eq. (8), where ${S}_{xy}$ denotes the score of the $y$th criterion in the $x$th comparison.

8 $${S}_{xy}={{d}_{xy}^{+}}^{^{\prime}}+{{d}_{xy}^{-}}^{^{\prime}}.$$

*Phase 5* Re-executing steps 3 and 4 until the number of remaining criteria reaches 2 for an even number of criteria or 1 for an odd number of criteria.

*Phase 6* Computing the weights of criteria according to Eqs. (9), (10), where ($\sum_{j=1}^{n}{W}_{j}=1)$

9 $${\mathbb{S}}_{j}=\sum_{y\in j}^{x}{S}_{xy},$$

10 $${W}_{j}=\sum_{j=1}^{y}{\mathbb{S}}_{j} y\in j. $$

## Material selection via SRP and VIMM: the first scenario methods

This section is divided into four sub-sections. In this first sub-section, the case study and the information on the materials, their properties, and the weights of the criteria for the evaluation of the materials are represented. In the second section, a group decision-making process conducted by seven experts is represented to select the vital and material criteria as the input for the VIMM method. VIMM method is applied to the case to compute the weights of criteria in the third section. The evaluation process of the material is represented in the fourth sub-section, in which the SRP method is applied to select the best material.

### Case study

A case study^96^ has been adopted in this paper to select the best phase change material to be used to store solar energy. Criteria taken in consideration are Latent Heat J/Kg ${(c}_{1})$, Density Kg/m3 ${(c}_{2})$, Specific Heat kJ/kg K ${(c}_{3})$, Specific Heat kJ/kg K ${(c}_{4})$, Thermal Conductivity W/m K ${(c}_{5})$ and Cost ${(c}_{6})$. Among all the criterion Latent Heat, Density, Specific Heat (solid), Specific Heat (liquid) and Thermal Conductivity are beneficial criterion i.e. higher the value better the alternative. Cost is non-beneficial criterion i.e. lower the value better the alternative. Nine alternatives are considered as the phase change material such as Calcium chloride hexa-hydrate (A1), Stearic acid (A2), p116 (A3), RT 60 (A4), Paraffin wax RT 30 (A5), n-Docosane (A6), n-Octadecane (A7), n-Nonadecane (A8) and n-Eicosane (A9). The information on the properties of the materials and the material selection’s decision matrix, including the materials and the criteria for the evaluation, is demonstrated in Tables 4 and 5 respectively. The weights of the criteria are also illustrated in Table 6.

**Table 4:** Properties of PCMs for solar energy devices.

Phase change material (PCM)	Material selection criteria					
	Latent heat J/Kg ${(c}_{1})$	Density Kg/m^3^ ${(c}_{2})$	Specific heat kJ/kg K (Cp(s))${(c}_{3})$	Specific heat kJ/kg K (Cp(l))${(c}_{4})$	Thermal conductivity W/m K ${(c}_{5})$	Cost ${(c}_{6})$
Calcium chloride hexahydrate (A1)	169.98	1560.0	1.4600	2.1300	1.0900	Very low
Stearic acid (A2)	186.50	903.00	2.8300	2.3800	0.1800	Very high
p116 (A3)	190.00	830.00	2.1000	2.1000	0.2100	Low
RT 60 (A4)	214.40	850.00	0.9000	0.9000	0.2000	Very low
Paraffin wax RT 30 (A5)	206.00	789.00	1.8000	2.4000	0.1800	Low
n-Docosane (A6)	194.60	785.00	1.9300	2.3800	0.2200	Low
n-Octadecane (A7)	245.00	773.22	0.3767	2.2670	0.1400	Low
n-Nonadecane (A8)	222.00	775.80	1.7189	1.9210	0.1420	High
n-Eicosane (A9)	247.00	776.33	0.7467	2.3770	0.1380	Low

**Table 5:** Material selection decision matrix.

PCM	Material selection criteria					
	${c}_{1}$	${c}_{2}$	${c}_{3}$	${c}_{4}$	${c}_{5}$	${c}_{6}$
A1	169.98	1560.0	1.4600	2.1300	1.*0*900	0.2550
A2	186.50	903.00	2.8300	2.3800	0.1800	0.7450
A3	190.00	830.00	2.1000	2.1000	0.2100	0.3350
A4	214.40	850.00	0.9000	0.9000	0.2000	0.2550
A5	206.00	789.00	1.8000	2.4000	0.1800	0.3350
A6	194.60	785.00	1.9300	2.3800	0.2200	0.3350
A7	245.00	773.22	0.3767	2.2670	0.1400	0.3350
A8	222.00	775.80	1.7189	1.9210	0.1420	0.6650
A9	247.00	776.33	0.7467	2.3770	0.1380	0.3350

**Table 6:** Criteria weights for the considered material selection case study.

Criteria	${c}_{1}$	${c}_{2}$	${c}_{3}$	${c}_{4}$	${c}_{5}$	${c}_{6}$
Weight	0.4901	0.1674	0.0528	0.0528	0.2109	0.0261

### The group decision-making process

In this section, in order to determine the vital and immaterial criteria as the main elements of VIMM algorithm, seven experts are employed to re-evaluate the material selection criteria weights. The original criteria weights are exhibited in Table 6.

The number of pairwise comparisons in VIMM for the even number of criteria is (n/2), where $n$ is the number of criteria. Therefore, in this case, there are two comparison processes. The seven experts are asked to select three criteria as the vital and immaterial criterion for each comparison based on the criteria priority of being the best choice to meet the properties of the vital or immaterial criteria. Table 7 demonstrates expert responses, where ${\mathbb{Z}}_{{\alpha }_{x}}^{y}$ and ${\mathbb{Z}}_{{\beta }_{x}}^{y}$ represent different choice of decision-makers in the selection of vital and immaterial criteria in each comparison, $y$ shows the priority of the selected criterion, $x$ indicates the number of comparison process, ${\mathbb{N}}$ is the set of natural numbers, and ${\psi }_{{\mathbb{Z}}_{{\alpha }_{x}}^{y}}$ and ${\psi }_{{\mathbb{Z}}_{{\beta }_{x}}^{y}}$ are the cardinal numbers, representing the frequency of the criteria that decision-makers selected as the vital and immaterial criteria respectively see Eqs. (11) and (12).

**Table 7:** Responses of decision-makers for selection of vital and immaterial criteria in the two comparison processes.

	${\alpha }_{1}$			${\beta }_{1}$			${\alpha }_{2}$			${\beta }_{2}$		
Expert	${\mathbb{Z}}_{{\alpha }_{1}}^{1}$	${\mathbb{Z}}_{{\alpha }_{1}}^{2}$	${\mathbb{Z}}_{{\alpha }_{1}}^{3}$	${\mathbb{Z}}_{{\beta }_{1}}^{1}$	${\mathbb{Z}}_{{\beta }_{1}}^{2}$	${\mathbb{Z}}_{{\beta }_{1}}^{3}$	${\mathbb{Z}}_{{\alpha }_{2}}^{1}$	${\mathbb{Z}}_{{\alpha }_{2}}^{2}$	${\mathbb{Z}}_{{\alpha }_{2}}^{3}$	${\mathbb{Z}}_{{\beta }_{2}}^{1}$	${\mathbb{Z}}_{{\beta }_{2}}^{2}$	${\mathbb{Z}}_{{\beta }_{2}}^{3}$
1	${c}_{1}$	${c}_{1}$	${c}_{1}$	${c}_{6}$	${c}_{6}$	${c}_{6}$	${c}_{5}$	${c}_{2}$	${c}_{2}$	${c}_{4}$	${c}_{4}$	${c}_{3}$
2	${c}_{1}$	${c}_{1}$	${c}_{5}$	${c}_{6}$	${c}_{3}$	${c}_{4}$	${c}_{5}$	${c}_{5}$	${c}_{2}$	${c}_{3}$	${c}_{4}$	${c}_{6}$
3	${c}_{5}$	${c}_{5}$	${c}_{1}$	${c}_{4}$	${c}_{6}$	${c}_{6}$	${c}_{1}$	${c}_{2}$	${c}_{5}$	${c}_{6}$	${c}_{4}$	${c}_{4}$
4	${c}_{1}$	${c}_{1}$	${c}_{2}$	${c}_{3}$	${c}_{6}$	${c}_{4}$	${c}_{2}$	${c}_{5}$	${c}_{5}$	${c}_{6}$	${c}_{4}$	${c}_{3}$
5	${c}_{1}$	${c}_{5}$	${c}_{2}$	${c}_{6}$	${c}_{6}$	${c}_{6}$	${c}_{1}$	${c}_{5}$	${c}_{5}$	${c}_{3}$	${c}_{4}$	${c}_{4}$
6	${c}_{1}$	${c}_{1}$	${c}_{5}$	${c}_{6}$	${c}_{6}$	${c}_{3}$	${c}_{5}$	${c}_{5}$	${c}_{5}$	${c}_{3}$	${c}_{3}$	${c}_{6}$
7	${c}_{1}$	${c}_{1}$	${c}_{1}$	${c}_{4}$	${c}_{6}$	${c}_{6}$	${c}_{5}$	${c}_{5}$	${c}_{5}$	${c}_{4}$	${c}_{3}$	${c}_{6}$

11 $${\psi }_{{\mathbb{Z}}_{{\alpha }_{x}}^{1}}\le {\psi }_{{\mathbb{Z}}_{{\alpha }_{x}}^{2}}\le {\psi }_{{\mathbb{Z}}_{{\alpha }_{x}}^{3}}, 1\le y\le 3, y\in {\mathbb{N}}, x=n/2,(n-1)/2, n\in j ,$$

12 $${\psi }_{{\mathbb{Z}}_{{\beta }_{x}}^{1}}\le {\psi }_{{\mathbb{Z}}_{{\beta }_{x}}^{2}}\le {\psi }_{{\mathbb{Z}}_{{\beta }_{x}}^{3}}, 1\le y\le 3, y\in {\mathbb{N}}, x=n/2,(n-1)/2, n\in j.$$

Table 8 shows the response distribution, where ${F}_{{c}_{j}}$ denotes the frequency of the criterion in each choice for the selection of the vital and immaterial criterion in the comparison processes. According to the distribution of responses of DMs (Table 8), the vital and immaterial selected by DMs criteria are shown in Tables 9 and 10.

**Table 8:** Distribution of decision-makers’ responses.

Criteria	${F}_{{c}_{j}}$											
	${\psi }_{{\mathbb{Z}}_{{\alpha }_{1}}^{1}}$	${\psi }_{{\mathbb{Z}}_{{\alpha }_{1}}^{2}}$	${\psi }_{{\mathbb{Z}}_{{\alpha }_{1}}^{3}}$	${\psi }_{{\mathbb{Z}}_{{\beta }_{1}}^{1}}$	${\psi }_{{\mathbb{Z}}_{{\beta }_{1}}^{2}}$	${\psi }_{{\mathbb{Z}}_{{\beta }_{1}}^{3}}$	${\psi }_{{\mathbb{Z}}_{{\alpha }_{2}}^{1}}$	${\psi }_{{\mathbb{Z}}_{{\alpha }_{2}}^{2}}$	${\psi }_{{\mathbb{Z}}_{{\alpha }_{2}}^{3}}$	${\psi }_{{\mathbb{Z}}_{{\beta }_{2}}^{1}}$	${\psi }_{{\mathbb{Z}}_{{\beta }_{2}}^{2}}$	${\psi }_{{\mathbb{Z}}_{{\beta }_{2}}^{3}}$
${c}_{1}$	6	5	3	0	0	0	2	0	0	0	0	0
${c}_{2}$	0	0	2	0	0	0	1	2	2	0	0	0
${c}_{3}$	0	0	0	1	1	1	0	0	0	3	2	2
${c}_{4}$	0	0	0	2	0	2	0	0	0	2	5	2
${c}_{5}$	1	2	2	0	0	0	4	5	5	0	0	0
${c}_{6}$	0	0	0	4	6	4	0	0	0	2	0	3

**Table 9:** Distribution of decision-makers’ responses regarding the vital criterion.

Criteria	${\psi }_{{\mathbb{Z}}_{{\alpha }_{1}}^{1}}$	${\psi }_{{\mathbb{Z}}_{{\alpha }_{1}}^{2}}$	${\psi }_{{\mathbb{Z}}_{{\alpha }_{1}}^{3}}$	${\psi }_{{\mathbb{Z}}_{{\alpha }_{2}}^{1}}$	${\psi }_{{\mathbb{Z}}_{{\alpha }_{2}}^{2}}$	${\psi }_{{\mathbb{Z}}_{{\alpha }_{2}}^{3}}$
${c}_{1}$	6	5	3	2	0	0
${c}_{2}$	0	0	2	1	2	2
${c}_{3}$	0	0	0	0	0	0
${c}_{4}$	0	0	0	0	0	0
${c}_{5}$	1	2	2	4	5	5
${c}_{6}$	0	0	4	0	0	0

**Table 10:** Distribution of decision-makers’ responses regarding the immaterial criterion.

Criteria	${\psi }_{{\mathbb{Z}}_{{\beta }_{1}}^{1}}$	${\psi }_{{\mathbb{Z}}_{{\beta }_{1}}^{2}}$	${\psi }_{{\mathbb{Z}}_{{\beta }_{1}}^{3}}$	${\psi }_{{\mathbb{Z}}_{{\beta }_{2}}^{1}}$	${\psi }_{{\mathbb{Z}}_{{\beta }_{2}}^{2}}$	${\psi }_{{\mathbb{Z}}_{{\beta }_{2}}^{3}}$
${c}_{1}$	0	0	0	0	0	0
${c}_{2}$	0	0	0	0	0	0
${c}_{3}$	1	1	1	3	2	2
${c}_{4}$	2	0	2	2	5	2
${c}_{5}$	0	0	0	0	0	0
${c}_{6}$	4	6	4	2	0	3

#### Determining the vital and immaterial criteria

The collected data in the previous section are evaluated in this section to determine the vital and immaterial criteria. The evaluation comprises two steps as follows:

*Step 1* The first step of the evaluation process of decision-makers’ opinions is to compute the value of frequency of vital and immaterial criteria. The step is conducted using Eqs. (13), (14), (15), (16) and (17).

13 $${V}_{{F}_{{c}_{j}}}^{{\alpha }_{x}}=\left(y\pm \xi \right){\psi }_{{\mathbb{Z}}_{{\alpha }_{x}}^{1}}+\left(\left(y-1\right)\pm \mu \right){\psi }_{{\mathbb{Z}}_{{\alpha }_{x}}^{2}}+\left(\left(y-2\right)\pm \nu \right){\psi }_{{\mathbb{Z}}_{{\alpha }_{x}}^{3}};$$

14 $${V}_{{F}_{{c}_{j}}}^{{\beta }_{x}}=\left(y\pm \xi \right){\psi }_{{\mathbb{Z}}_{{\beta }_{1}}^{1}}+\left(\left(y-1\right)\pm \mu \right){\psi }_{{\mathbb{Z}}_{{\beta }_{1}}^{2}}+\left(\left(y-2\right)\pm \nu \right){\psi }_{{\mathbb{Z}}_{{\beta }_{1}}^{3}};$$

15 $$\xi =\left\{\begin{array}{c}0\le \xi \le y, y+\xi \\ 0<\xi , y-\xi \end{array}\right.;$$

16 $$\mu =\left\{\begin{array}{c}0\le \mu \le y, \left(y-1\right)-\mu \\ y>\mu +1, \left(y-1\right)-\mu \end{array}\right.;$$

17 $$\nu =\left\{\begin{array}{c}0\le \nu \le y, \left(y-2\right)+\nu \\ y>\nu +2, \left(y-2\right)-\nu \end{array}\right.;$$

where the values of $\xi$, $\mu$, and $\nu$ are determined by decision-makers.

*Step 2* The second step is to determine the vital and immaterial criteria in each comparison which is computed with the selection of the maximum value of Eqs. (13), (14), where.$\underset{1\le j\le 6}{\mathit{max}}{V}_{{F}_{{c}_{j}}}^{{\alpha }_{x}}$ and $\underset{1\le j\le 6}{\mathit{max}}{V}_{{F}_{{c}_{j}}}^{{\beta }_{x}}$ are the vital and immaterial in $\mathrm{x}$th comparison.

In this case, we consider $\xi ,\mu ,\nu =0$ for the selection of the immaterial and vital criteria. According to Eqs. (13) and (14), results of the evaluation of the decision-makers’ opinions are demonstrated in Tables 11 and 12 where ${c}_{1}$ (Latent Heat) is determined as the vital in the first comparison, and ${c}_{5}$ (Thermal Conductivity) is determined as the second vital criterion in the second comparison. Criterion ${c}_{6}$ (Cost) plays the immaterial criterion role and ${c}_{4}$ (Specific Heat) is selected by the experts to affect the criteria weighting process as the second immaterial criterion in the second comparison, as shown in Table 12 of the first comparison.

**Table 11:**
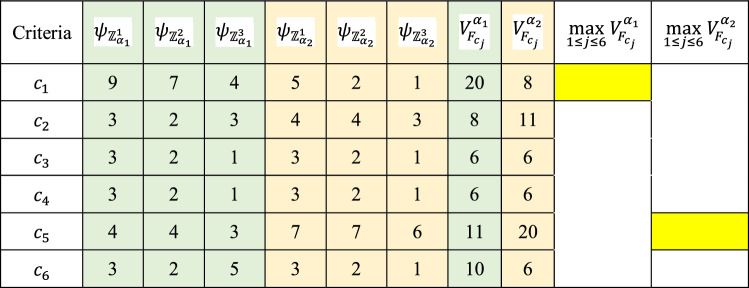
The vital criteria selection for two comparison processes based on expert opinions.

**Table 12:**
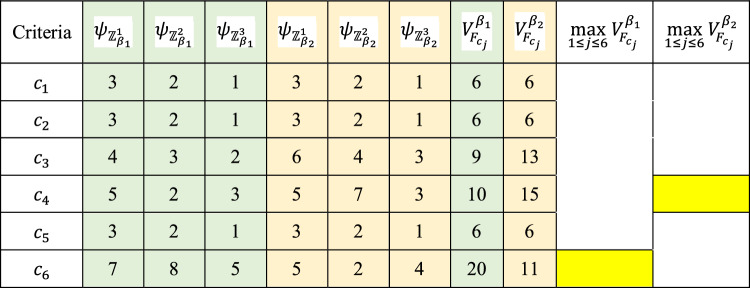
The immaterial criteria selection for two comparison processes based on the experts’ opinion.

### Computing weights of criteria

The prerequisite of VIMM algorithm is the goal definition, where if there is one goal for the decision-making problem, the first scenario ought to be employed; otherwise, VIMM: the second scenario must be used. This paper’s case follows one goal: selecting the most proper phase change material to store solar energy.

Since the weights were computed in^96^ work, in contrast to the classic VIMM and instead of using the algorithm for the computation of the vital and immaterial criteria, we relied on the experts’ opinions and the vital and immaterial criteria in the second comparison are selected by them. In our case, there are no mediocre criteria selected by decision-makers.

The first step of computing weights of criteria is to allocate 5 and 1 to the first selected vital and immaterial criteria which are ${c}_{1}$, the Latent Heat, and ${c}_{6}$, the Cost, then:

$${c}_{1}=5$$

$${c}_{6}=1$$

The seven experts are asked to give their expert opinions to run the comparison. The first two comparisons of the remaining criteria and the selected vital and immaterial are shown in Tables 13 and 14, where the scale as illustrated in Fig. 2 is used to conduct the comparison. The next phase is to compute the distances between all criteria, and the vital and immaterial criteria are shown in Table 15, where ${d}^{+}$ stands for the distance between the criteria and the immaterial criterion, while ${d}^{-}$ displays the distance between the criteria and the vital criterion. To display the computation of ${d}^{+}$ and ${d}^{-}$, the following examples are provided. The normalized distance matrix, as shown in Table 16, is derived using Eqs. 6, and 7, where $d^{ + \prime }$ and $d^{ - \prime }$ indicate the normalized distances.

**Table 13:** Comparison of latent heat (vital criterion) with other criteria.

Criteria	${c}_{2}$	${c}_{3}$	${c}_{4}$	${c}_{5}$
${c}_{1}$	6.166	3.882	3.827	7.053

**Table 14:** Comparison of cost (immaterial criterion) against other criteria.

Criteria	${c}_{2}$	${c}_{3}$	${c}_{4}$	${c}_{5}$
${c}_{6}$	6.414	2.126	2.023	8.080

**Table 15:** Distances between the criteria and first vital and immaterial criteria.

Criteria	$d^{ + \prime }$	$d^{ - \prime }$
${c}_{2}$	5.414	3.834
${c}_{3}$	1.126	6.118
${c}_{4}$	1.023	6.173
${c}_{5}$	7.080	2.947
Max	7.080	
Min		6.173

**Table 16:** Normalized distance matrix.

Criteria	${d}^{+}$	${d}^{-}$
${c}_{2}$	0.765	1.610
${c}_{3}$	0.159	1.009
${c}_{4}$	0.144	1.000
${c}_{5}$	1.000	2.095

$${d}_{\mathrm{Density}}^{+}=6.414-1$$

$${d}_{\mathrm{Density }}^{-}=10-6.166$$

The next step is to compute the first criteria’ scores and identify the second vital and immaterial using the VIMM algorithm’s “step no 4.2” and Eq. (8).

Similar to what have been selected by decision-makers (shown in Tables 11 and 12), as displayed in Table 17, Specific Heat and Thermal Conductivity are calculated as the immaterial and vital criteria, respectively. In the next step, the remaining criteria are compared with the second vital and immaterial criteria, the Thermal Conductivity and the Specific Heat. The comparisons are conducted by decision-makers as presented in Tables 16 and 17, and the corresponding scores are given in Tables 18 and 19, and the normalized distance matrix is illustrated in Table 20.

**Table 17:**
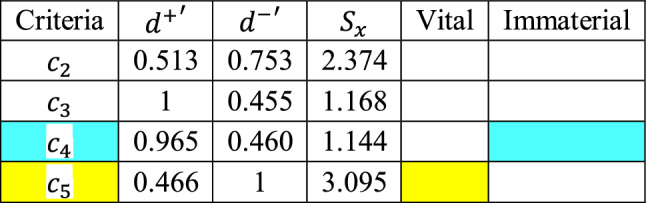
The first scores, and the second vital and immaterial criteria.

**Table 18:** Comparison of the vital criterion ${c}_{5}$(thermal conductivity) with the remaining criteria.

Criteria	${c}_{2}$	${c}_{3}$
${c}_{5}$	0.1674	0.0555

**Table 19:** Comparison of the immaterial criterion, ${c}_{4} ($specific heat, (Cp(l)), against the remaining criteria.

Criteria	${c}_{2}$	${c}_{3}$
${c}_{4}$	0.1674	0.0555

**Table 20:**
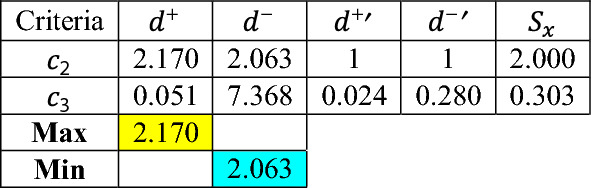
Normalized distance matrix and second scores of criteria.

The final phase is the computation of the weights, which are extracted from the scores. To do that, first, the scores need to be calculated. The final comparison is shown in (Table 21) in which Density is the final vital criterion and the Specific Heat (Cp(s)) is the last immaterial criterion. The scoring continues until all criteria received their final 5 and 1 values. The allocation of value to the vital criteria continues to the last comparison, while the immaterial criteria receive merely one time their corresponding value. The criteria weights as derived using Eqs. (9) and (10) are the displayed in Table 22.

**Table 21:**
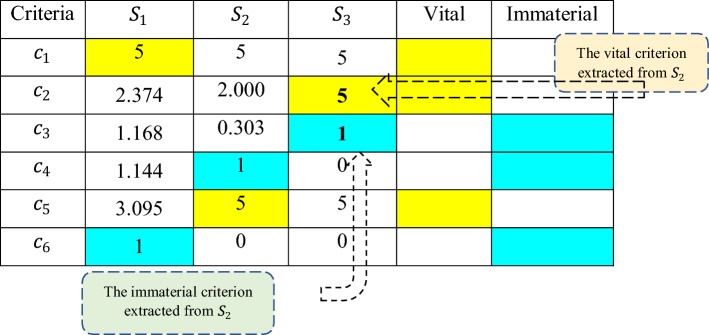
Final comparison process.

Significant values are in bold.

**Table 22:**
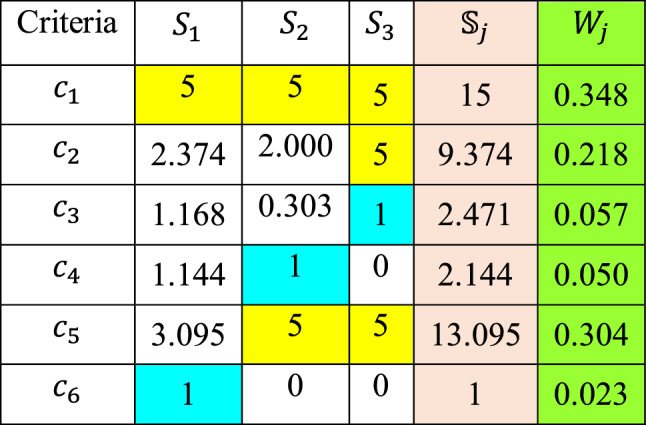
Derived criteria weights.

### Ranking materials

The final step in evaluating the materials is to derive the ranking orders of the alternatives using SRP which is based on ranking each alternative in relation to each criterion. The weighted ranking matrix, obtained from the material evaluation decision matrix of Table 5 using Eqs. (2) and (3), is shown in Table 23. The ranking order, as presented in Table 24, is determined using the total ranking scores of the alternatives, calculated in the fifth stage of SRP method using Eq. (4). The ranks of the materials are finally shown in the same table employing Eq. (6) which indicates RT 60 (A4) as the best material to choose for the considered application.

**Table 23:** Weighted ranking matrix using group decision-making and VIMM: the first scenario.

${W}_{j}$	0.348	0.218	0.057	0.050	0.304	0.023						
PCM	${c}_{1}$	${c}_{2}$	${c}_{3}$	${c}_{4}$	${c}_{5}$	${c}_{6}$	${c}_{1}$	${c}_{2}$	${c}_{3}$	${c}_{4}$	${c}_{5}$	${c}_{6}$
A1	9	1	6	5	1	1	3.132	0.218	0.342	0.25	0.304	0.023
A2	8	2	1	2	5	5	2.784	0.436	0.057	0.1	1.52	0.115
A3	7	4	2	6	3	2	2.436	0.872	0.114	0.3	0.912	0.046
A4	4	3	7	8	4	1	1.392	0.654	0.399	0.4	1.216	0.023
A5	5	5	4	1	5	2	1.74	1.09	0.228	0.05	1.52	0.046
A6	6	6	3	2	2	2	2.088	1.308	0.171	0.1	0.608	0.046
A7	2	9	9	4	7	2	0.696	1.962	0.513	0.2	2.128	0.046
A8	3	8	5	7	6	4	1.044	1.744	0.285	0.35	1.824	0.092
A9	1	7	8	3	8	3	0.348	1.526	0.456	0.15	2.432	0.069

**Table 24:** Total ranking scores of materials along with derived ranking.

PCM	${SR}_{i}$	${\mathfrak{R}}_{i}$	Rank by SRP
A1	4.269	4.731	2
A2	5.012	3.988	7
A3	4.68	4.32	5
A4	4.084	4.916	1
A5	4.674	4.326	4
A6	4.321	4.679	3
A7	5.545	3.455	9
A8	5.339	3.661	8
A9	4.981	4.019	6

## Discussion

This paper introduced SRP to solve material selection problems. In this new method, the importance weights of criteria play the leading role in evaluating the decision-making problems' alternatives. Since the new method algorithm's process is mainly grounded on the ranks of each alternative against each criterion, it heavily relies on the weights of each criterion.

SRP is applied to a material selection problem to evaluate a set of materials used to store solar energy. Although the criteria weights have already been determined by Rathod and Kanzaria^96^, yet we asked seven experts to re-evaluate the criteria. VIMM, a reliable MCDM subjective weighting method, is used for extracting opinions of the decision-makers. The original criteria weights, as determined by Rathod and Kanzaria^96^, are also utilized to compare the differences between the produced results in order to show how sensitive the SRP is to the criteria weights (see Table 25).

**Table 25:** New ranking orders obtained using the original weights, estimated by Rathod and Kanzaria^96^.

${W}_{j}$	0.4901	0.1674	0.0528	0.0528	0.2109	0.0261	SRP calculations		
PCM	${c}_{1}$	${c}_{2}$	${c}_{3}$	${c}_{4}$	${c}_{5}$	${c}_{6}$	${SR}_{i}$	${\mathfrak{R}}_{i}$	Ranking
A1	9	1	6	5	1	1	5.3961	3.6039	9
A2	8	2	1	2	5	5	5.599	3.401	8
A3	7	4	2	6	3	2	5.2076	3.7924	7
A4	4	3	7	8	4	1	4.1243	4.8757	2
A5	5	5	4	1	5	2	4.6582	4.3418	3
A6	6	6	3	2	2	2	4.683	4.317	4
A7	2	9	9	4	7	2	4.7017	4.2983	5
A8	3	8	5	7	6	4	4.8129	4.1871	6
A9	1	7	8	3	8	3	4.0082	4.9918	1

**Figure 4:**
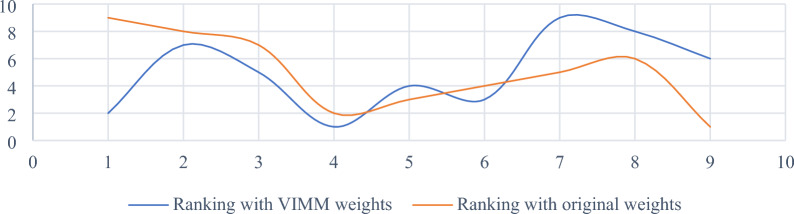
Comparative analysis of rankings affected by the criteria weights computed by the VIMM method and the original criteria weights.

The difference between the two rankings is illustrated in Fig. 4. To better understand the sensitivity of SRP to criteria weights, different weight sets computed by two most popular MCDM objective weighting methods namely Shannon’s entropy and CRITIC are considered here. The set of weights computed by Entropy and CRITIC methods are as follows:

$${W}_{j}^{entropy}=\left\{\mathrm{0.012,0.050,0.194,0.046,0.571,0.128}\right\},$$

$${W}_{j}^{CRITIC}=\left\{\mathrm{0.227,0.134,0.176,0.151,0.131,0.180}\right\}.$$

**Figure 5:**
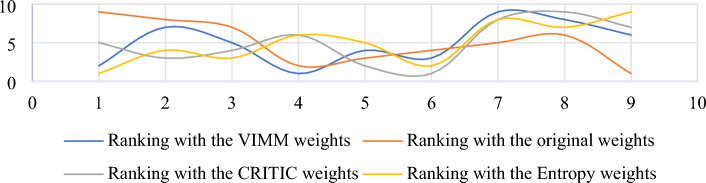
Comparative analysis of rankings affected by different weights computed by VIMM, CRITIC, Entropy, and original methods.

Figure 5 shows considerable differences between the ranks of the results affected by distinctive weights derived by different MCDM weighting methods, including objective and subjective approaches. This figure also demonstrates high sensitivity of SRP to criteria weighting. Reliability of SRP is directly related to the reliability of weighting method used to assess the criteria under investigation. In this paper, VIMM method is used as a subjective weighting tool to derive criteria weights from decision-makers’ opinions. This goal-oriented MCDM weighting method is more reliable than classic forms of BWM and AHP. According to Zakeri et al.^9^, there are three main advantages of VIMM over the mentioned weighting methods including fewer number pairwise comparison, where $n(n-1)/2$ and $2n-3$ number of comparisons are required for AHP and BWM respectively, while VIMM needs merely $(n-1)/2$ and $n/2$ number of comparisons for even and odd numbers of criteria in the evaluation process. In contrast to AHP method, VIMM is not limited to the number of criteria since it re-evaluates the criteria in every process. VIMM is designed to consider the decision-making goal(s) by proposing two scenarios, where the first scenario is developed to evaluate the weights of criteria in a decision-making problem with one goal, and the second scenario is developed to consider more than one goal in its process of the computation of the criteria weights. Thus, it is a proper pair for SRP in ranking alternatives to a decision-making problem.

**Figure 6:**
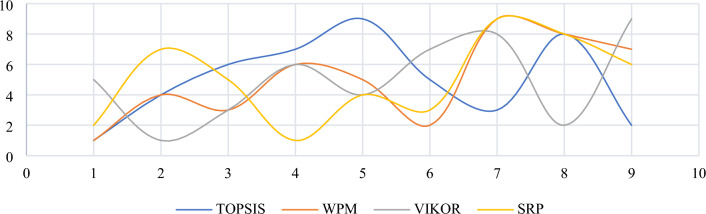
Comparison between rankings generated by four MCDM methods.

Four MCDM methods, including WPM, VIKOR, and TOPSIS have also been applied to compare the results obtained from SRP. The difference between rankings is pictured in Fig. 6.

Figure 6 demonstrates that there is no compromise in ranking among the four methods. Thus, the comparison of MCDM results in the previous section cannot be used to validate the findings, leading to the next section.

### The compromise decision index (CDI)

In complex MCDM problems with several alternatives and criteria, irregularity in the result of comparing the generated rankings is inevitable. As shown in the previous section, there is an irregularity in the rankings generated by the MCDM methods; therefore, for these types of situations, we have proposed an index called *CDI* to interpret the comparison between the outputs of MCDM methods in the processes that at least four MCDM methods are applied to solve an MCDM problem (${\rm K}\ge 4$), where ${\rm K}$ denotes the number of MCDM methods, the number of alternatives equals $m\ge 2{\rm K}$, and the number of criteria equals $n\ge (2k-2)$, which is the limitation of AHP in the evaluation of criteria where it is restricted to seven (+ ⁄– 2) criteria^9^.

The following steps shows the computation process of *CDI*.

*Step 1* Computing the performance of each alternative in accordance with Eqs. (18) and (19) respectively, where ${\delta }_{i}$ is the performance of $i$th alternative, ${\zeta }_{\mathbb{R}}$ denotes weight of each rank, ${\mathbb{R}}$ stands for each rank, and ${A}_{i}$ states the $i$th alternative.

18 $${\delta }_{i}={\zeta }_{{\mathbb{R}}_{i}}{F}_{{A}_{i}}^{{\mathbb{R}}_{i}}, i=\left\{\mathrm{1,2},\dots ,m\right\}, {F}_{{A}_{i}}^{{\mathbb{R}}_{i}}\le {\rm K},$$

19 $${\zeta }_{{\mathbb{R}}_{i}}={\left(\sum {\zeta }_{{\mathbb{R}}_{i}}\right)}^{-1}\left(m-{\mathbb{R}}_{i}+1\right), $$

*Step 2* Ranking alternatives according to the higher value of ${\delta }_{i}$.

*Step 3* Calculating the deviation of each ranking fashioned by each MCDM method to the performance of alternative, according to (Eq. (20)).

20 $${\sigma }_{i}=\sqrt{\sum_{K=1}^{z}{\left({X}_{i}-{Y}_{i}^{K}\right)}^{2}}, K=\left\{1,..,z\right\}, i=\left\{1,\dots ,m\right\},$$

*Step 4* The final step is the computation of *CDI* values using Eq. (21).

21 $$CDI=1/100\sum_{i=1}^{m}{\sigma }_{i}.$$

*CDI* puts the results of MCDM methods in four types of compromises: Pragmatic compromise, Rational compromise, Fair compromise, and Rotten compromise. The results are interpreted concerning these compromises according to the defined categories provided by Wendt^97^.

#### The pragmatic compromise

When there is a pragmatic reason for compromise, the pragmatic compromise makes. When *CDI* shows the ranks have pragmatic compromise, results of the MCDM method are considered to be reliable.

#### The rational compromise

According to Wendt.^97^, a compromise is rational when it is rational for all parties to agree on the compromise. When *CDI* interprets the comparison of ranks as the rational compromise, ranking produced by the MCDM method is reliable to some extent. However, it is better to add another MCDM method to reach a pragmatic compromise.

#### The fair compromise

When a fair compromise is made in the comparison analysis, decision-makers could decide whether to consider the compromise as a rational compromise or rely on the practical results.

#### The rotten compromise

According to Wendt.^97^, the purpose of introducing the concept of a "rotten compromise" is to have a term that signifies compromises that are morally dubious or unethical. When *CDI* interprets the results as a rotten compromise, it means that the results of MCDM methods cannot be validated theoretically, and practical results must be evaluated and validated.

**Figure 7:**
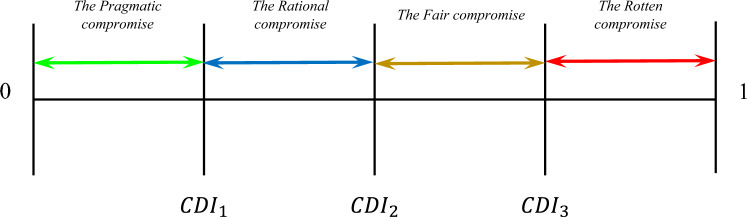
Interpretation of the MCDM results comparison based on the *CDI*.

To interpret the results in accordance with the categories, first the maximum deviation needs to be computed. The minimum deviation equals to zero, where all the applied MCDM methods generated the same results. The interpretation of the results is based on Fig. 7, where

22 $${CDI}_{1}=\underset{1\le i\le m}{\mathrm{max}}{\sigma }_{i}/4,$$

23 $${CDI}_{2}=\underset{1\le i\le m}{\mathrm{max}}{\sigma }_{i}/2, $$

24 $${CDI}_{3}=\underset{1\le i\le m}{\mathrm{max}}{\sigma }_{i}.$$

**Figure 8:**
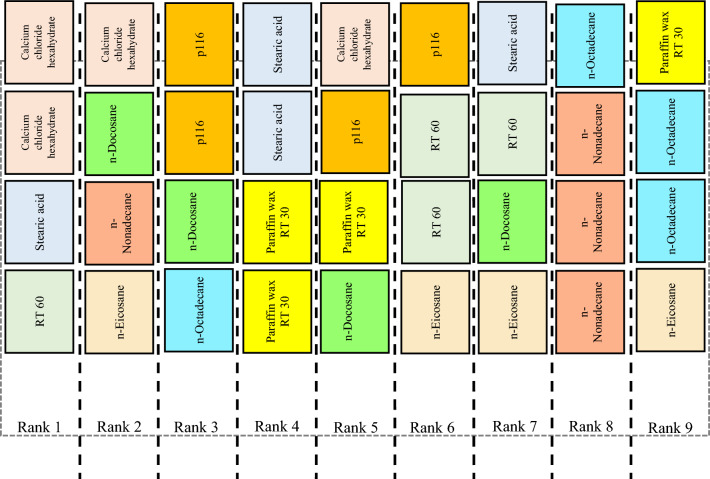
Distribution of each material rank according to the MCDM methods.

**Figure 9:**
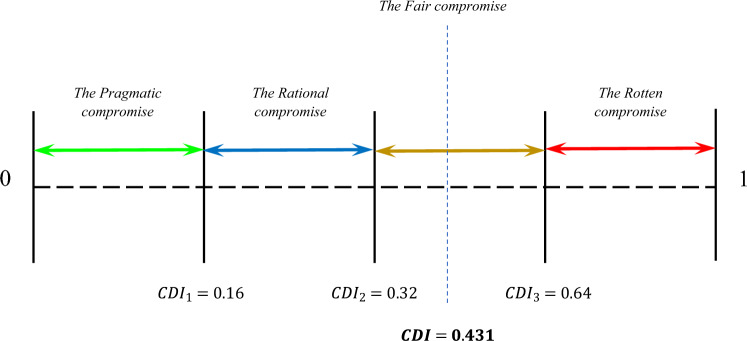
The fair compromise of *CDI* for the material selection problem.

To interpret the obtained results from the comparison between the MCDM results with *CDI*, the distribution of the materials’ rankings generated by the four MCDM methods needs to be calculated (see Fig. 8). According to Eqs. (18) and (19), performance of the materials is shown in Table 26. According to Eqs. (20), (21), $CDI=$ 0.430705. According to Eqs. (22), (23), (24), the interpretation of *CDI* is the fair compromise (see Fig. 9), where $\underset{1\le i\le m}{\mathrm{max}}{\sigma }_{i}=0.64$.

**Table 26:** Performance of materials.

${\zeta }_{{\mathbb{R}}_{i}}$	${\mathbb{R}}_{1}$	${\mathbb{R}}_{2}$	${\mathbb{R}}_{3}$	${\mathbb{R}}_{4}$	${\mathbb{R}}_{5}$	${\mathbb{R}}_{6}$	${\mathbb{R}}_{7}$	${\mathbb{R}}_{8}$	${\mathbb{R}}_{9}$	${\delta }_{i}$	Rank
	0.200	0.178	0.156	0.133	0.111	0.089	0.067	0.044	0.022		
PCM	${F}_{{A}_{1}}^{{\mathbb{R}}_{1}}$	${F}_{{A}_{2}}^{{\mathbb{R}}_{2}}$	${F}_{{A}_{3}}^{{\mathbb{R}}_{3}}$	${F}_{{A}_{4}}^{{\mathbb{R}}_{4}}$	${F}_{{A}_{5}}^{{\mathbb{R}}_{5}}$	${F}_{{A}_{6}}^{{\mathbb{R}}_{6}}$	${F}_{{A}_{7}}^{{\mathbb{R}}_{7}}$	${F}_{{A}_{8}}^{{\mathbb{R}}_{8}}$	${F}_{{A}_{9}}^{{\mathbb{R}}_{9}}$		
A1	2	1	0	0	1	0	0	0	0	0.689	1
A2	1	0	0	2	0	0	1	0	0	0.533	2
A3	0	0	2	0	1	1	0	0	0	0.511	3
A4	1	0	0	0	0	2	1	0	0	0.444	4
A5	0	0	0	2	1	0	0	0	1	0.400	5
A6	0	1	1	0	1	0	1	0	0	0.511	3
A7	0		1	0	0	0	0	1	2	0.244	8
A8	0	1	0	0	0	0	0	3	0	0.311	7
A9	0	1	0	0	0	1	1	0	1	0.356	6

$${CDI}_{1}=0.16$$

$${CDI}_{2}=0.32$$

$${CDI}_{3}=0.64$$

### The comparative rankings analysis -the performance analysis

**Figure 10:**
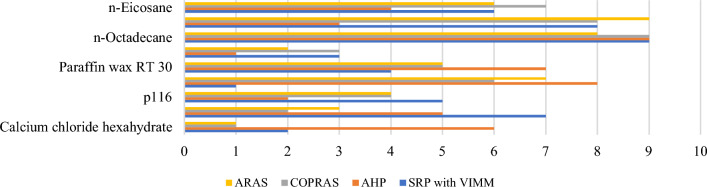
Ranking of alternative materials using different MCDM methods.

Similar to SRP, AHP is also highly sensitive to criteria weights. This section uses the Zakeri–Konstantas performance correlation coefficient and the dependency analysis to evaluate the SRP performance. ARAS and COPRAS (see Goswami et al.^6^ are also added to the list for the similarity evaluation. The rankings generated by the mentioned methods are illustrated in Fig. 10. The Zakeri-Konstantas performance correlation coefficient has been employed to execute the comparative performance analysis of the MCDM methods, and the dependency analysis is used to validate the new MCDM method.

#### Zakeri-Konstantas performance correlation coefficient

**Figure 11:**
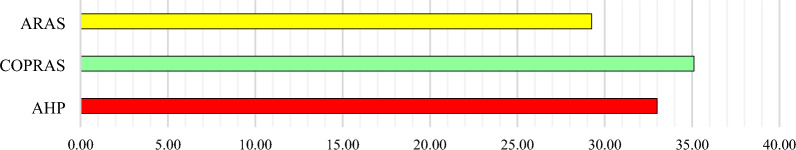
Performance similarity evaluation of ARPAS, COPRAS, and AHP with SRP using Zakeri-Konstantas performance correlation coefficient.

Zakeri-Konstantas performance correlation coefficient a new tool to evaluate the similarities of the outputs of the MCDM methods. Introduced by^98^, Zakeri–Konstantas performance correlation coefficient is a new nonparametric measure of rank correlation that measures the similarities between the rankings generated by different MCDM methods. In order to provide the similarity degree of the two MCDM methods, the new coefficient computes each decision option's performance based on its corresponding rank in the data sets generated by the two MCDM methods. Equations (24) and (25) depict the computation of similarity conducted by Zakeri-Konstantas performance correlation coefficient, where $\mathrm{\rm Z}\mathrm{\rm K}$ stands for the Zakeri-Konstantas performance correlation coefficient, ${\mathbb{N}}$ denotes the natural numbers, and ${R}_{i}^{l}$ and ${R}_{i}^{h}$ show the rank of $i$th alternative generated by $l$th MCDM algorithm and the rank of $i$ th alternative generated by $h$th MCDM algorithm respectively. Zakeri-Konstantas performance correlation coefficient is architected on two main bases, 1. significance of each option using Eqs. (26),and (2). performance of each option in each rank using Eq. (27), and the total performance using Eq. (28). The performance analysis results are exhibited in Fig. 11, where COPRAS showed the most similar results to SRP, and ARAS has the slightest similarity in the performance according to the results obtained from the Zakeri-Konstantas performance correlation coefficient. The results also show that AHP and SRP are different in generating results.

25 $${\mathrm{\rm Z}\mathrm{\rm K}}_{\left(l:h\right)}=\frac{100}{m}\sum_{i=1}^{m}\frac{\underset{1\le i\le m}{\mathrm{min}}\left(\frac{{m}^{2}+m-m{R}_{i}^{l}}{{R}_{i}^{l}\sum_{i=1}^{m}i};\frac{{m}^{2}+m-m{R}_{i}^{h}}{{R}_{i}^{h}\sum_{i=1}^{m}i}\right)}{\underset{1\le i\le m}{\mathrm{max}}\left(\frac{{m}^{2}+m-m{R}_{i}^{l}}{{R}_{i}^{l}\sum_{i=1}^{m}i};\frac{{m}^{2}+m-m{R}_{i}^{h}}{{R}_{i}^{h}\sum_{i=1}^{m}i}\right)},$$

where

26 $${\mathrm{\rm Z}\mathrm{\rm K}}_{\left(l:h\right)}=\left\{\begin{array}{c}0, \sum_{i=1}^{m}{\langle {\left({R}_{i}^{l}-{R}_{i}^{h}\right)}^{2}\rangle }^{0.5}=\frac{{m}^{2}}{2}, m=2\kappa , \kappa \in N, \kappa \ne \mathrm{0,1} \\ 0, \sum_{i=1}^{m}{\langle {\left({R}_{i}^{l}-{R}_{i}^{h}\right)}^{2}\rangle }^{0.5}=\frac{{m}^{2}-1}{2}, m=2\kappa +1, \kappa \in N, \kappa \ne 0 \end{array},\right.$$

27 $${U}_{i}^{F}=\left(\left(m+1\right)-{R}_{i}^{F}\right){\sum_{i=1}^{m}i}^{-1}, i=\left\{1,\dots ,m\right\},$$

28 $${\Gamma }_{i}^{F}={R}_{i}^{F}\langle \left(\left(m+1\right)-{R}_{i}^{F}\right){\sum_{i=1}^{m}i}^{-1}\rangle , i=\left\{1,\dots ,m\right\},$$

29 $${\mathrm{\rm Z}\mathrm{\rm K}}_{\left(l:h\right)}^{t}={\mathrm{\rm Z}\mathrm{\rm K}}_{\left(l:h\right)}{(m-1)}^{-1}, i=\left\{1,\dots ,m\right\},$$

#### The dependency analysis

The comparison process was conducted in the previous section to determine how dependent each method is on the weights of criteria. As a result, a new statistical measure called dependency analysis was proposed instead of using sensitivity analysis. The dependency analysis measures the dependency of a ranking on the information embedded in a criterion's corresponding data set. Greater dependency leads to an increased sensitivity of the method to changes in criteria weights, which in turn results in greater reliability. The dependency analysis is grounded on three central concepts: fair vital importance, real importance, and fair feeble importance. It measures changes in a ranking based on the impact of the mentioned concepts to estimate the dependency of a ranking on each criterion. The following equations show its process, where ${\theta }_{j}^{{c}_{x}}$, ${\lambda }_{j}^{{c}_{x}}$, ${\beta }_{j}^{{c}_{x}}$, and ${w}_{j}^{{c}_{F}}$ stand for the real importance, the fair vital importance, fair feeble importance of $x$th criteria, and weights of the rest criteria, respectively. The constant values of ${\lambda }_{j}^{{c}_{x}}$, ${\beta }_{j}^{{c}_{x}}$, and ${w}_{j}^{{c}_{F}}$ have been provided in Tables 27 and 28.

**Table 27:** Constant values of the fair vital importance based on the number of criteria, 20 criteria, $\underset{1\le j\le n}{\mathrm{max}}j=20$.

	$$n$$															
	5	6	7	8	9	10	11	12	13	14	15	16	17	18	19	20
${\lambda }_{j}^{{c}_{x}}$	0.800	0.833	0.857	0.875	0.889	0.900	0.909	0.917	0.923	0.929	0.933	0.938	0.941	0.944	0.947	0.950
${w}_{j}^{{c}_{F}}$	0.050	0.033	0.024	0.018	0.014	0.011	0.009	0.008	0.006	0.005	0.005	0.004	0.004	0.003	0.003	0.003
$\varsigma$			$0.01\le \varsigma \le 0.05$			$0.05\le \varsigma \le 0.1$						$0.06\le \varsigma \le 0.11$

**Table 28:** Constant values of the fair feeble importance based on the number of criteria, 20 criteria, $\underset{1\le j\le n}{\mathrm{max}}j=20$.

	$$n$$															
	5	6	7	8	9	10	11	12	13	14	15	16	17	18	19	20
${\beta }_{j}^{{c}_{x}}$	0.250	0.217	0.193	0.175	0.161	0.150	0.081	0.073	0.067	0.061	0.057	0.053	0.049	0.046	0.043	0.040
${w}_{j}^{{c}_{F}}$	0.188	0.157	0.135	0.118	0.105	0.094	0.092	0.084	0.078	0.072	0.067	0.063	0.059	0.056	0.053	0.051
$\xi$	$0<\xi \le 0.05$						$0<\xi \le 0.025$									

In Eqs. (33) and (34), ${R}_{{A}_{i}}^{{\beta }_{j}^{{c}_{x}}}$, ${R}_{{A}_{i}}^{{\lambda }_{j}^{{c}_{x}}}$, and ${R}_{{A}_{i}}^{{\theta }_{j}^{{c}_{x}}}$ denote the rank $i$th alternative (${A}_{i}$), affected by the fair vital importance, fair feeble importance, and the real importance. ${\Omega }_{{c}_{x}}$ also demonstrates the overall dependency of the rank generated by an MCDM method to a criterion. $\Omega$ stands for the dependency of a ranking to the criteria, where $\Omega \le 0.5$ expresses the reliability of the generator, in our case an MCDM method. In fact, in a comparison process of the generators, MCDM methods, the one that its corresponding $\Omega$ is closer to 1 shows the more reliability and the more dependency to the criteria. In contrast, $\Omega \ge 0.5$ portrays the unreliability of the generator, or the MCDM method in the evaluation of the alternatives.

30 $${\theta }_{j}^{{c}_{x}}={w}_{x}, j=\left\{1,\dots ,n\right\}, x\in j, x\le n,$$

31 $${\lambda }_{j}^{{c}_{x}}=\left\{\begin{array}{l}{n}^{-1}\left(n-1\right)\sum {w}_{j}, j=\left\{1,\dots ,n\right\}, x\in j, x\le n, n\le 6 \\ \begin{array}{l}\langle {n}^{-1}\left(n-1\right)\sum {w}_{j}\rangle -\varsigma , j=\left\{1,\dots ,n\right\}, x\in j, x\le n, 7\le n\le 9, 0.01\le \varsigma \le 0.05 \\ \langle {n}^{-1}\left(n-1\right)\sum {w}_{j}\rangle -\varsigma , j=\left\{1,\dots ,n\right\}, x\in j, x\le n, 10\le n\le 15, 0.06\le \varsigma \le 0.09 \\ \langle {n}^{-1}\left(n-1\right)\sum {w}_{j}\rangle -\varsigma , j=\left\{1,\dots ,n\right\}, x\in j, x\le n, 15\le n\le 20, 0.06\le \varsigma \le 0.11\end{array}\end{array}\right.,$$

32 $${\beta }_{j}^{{c}_{x}}=\left\{\begin{array}{l}\langle {n}^{-1}\sum {w}_{j}\rangle +\xi , j=\left\{1,\dots ,n\right\}, x\in j, 0<\xi \le 0.05, x\le n, 5\le n\le 10 \\ \langle {n}^{-1}\sum {w}_{j}\rangle +\xi , j=\left\{1,\dots ,n\right\}, x\in j, 0<\xi \le 0.025, x\le n, 11\le n\le 20\end{array}\right.,$$

33 $${w}_{j}^{{c}_{F}}=\left\{\begin{array}{c}\frac{1-{\lambda }_{j}^{{c}_{x}}}{n-1}, j=\left\{1,\dots ,n\right\}, x\in j, x\le n, F=j-\left\{x\right\}\\ \frac{1-{\beta }_{j}^{{c}_{x}}}{n-1}, j=\left\{1,\dots ,n\right\}, x\in j, x\le n, F=j-\left\{x\right\}\end{array},\right.$$

34 $${\Omega }_{{c}_{j}}^{{A}_{i}}=\frac{{R}_{{A}_{i}}^{{\beta }_{j}^{{c}_{x}}}-{R}_{{A}_{i}}^{{\lambda }_{j}^{{c}_{x}}}}{{R}_{{A}_{i}}^{{\theta }_{j}^{{c}_{x}}}}\times 100, i=\left\{1,\dots ,m\right\}, j=\left\{1,\dots ,n\right\}, x\in n, F=j-\left\{x\right\},$$

Proof:

35 $${\Omega }_{{c}_{j}}^{{A}_{i}}=\langle \frac{{R}_{{A}_{i}}^{{\theta }_{j}^{{c}_{x}}}-{R}_{{A}_{i}}^{{\lambda }_{j}^{{c}_{x}}}}{{R}_{{A}_{i}}^{{\theta }_{j}^{{c}_{x}}}}\times 100\rangle -\langle \frac{{R}_{{A}_{i}}^{{\theta }_{j}^{{c}_{x}}}-{R}_{{A}_{i}}^{{\beta }_{j}^{{c}_{x}}}}{{R}_{{A}_{i}}^{{\theta }_{j}^{{c}_{x}}}}\times 100\rangle =\frac{{R}_{{A}_{i}}^{{\beta }_{j}^{{c}_{x}}}-{R}_{{A}_{i}}^{{\lambda }_{j}^{{c}_{x}}}}{{R}_{{A}_{i}}^{{\theta }_{j}^{{c}_{x}}}}\times 100,$$

36 $$\Omega ={n}^{-1}\sum n\langle \underset{1\le i\le m}{\mathrm{max}}{\sum_{j=1}^{n}{{\Omega }_{{c}_{j}}^{{A}_{i}}}^{2}}^{0.5}\rangle -{\sum_{j=1}^{n}{{\Omega }_{{c}_{j}}^{{A}_{i}}}^{2}}^{0.5}, 0\le\Omega \le 1, i=\left\{1,\dots ,m\right\},$$

**Figure 12:**
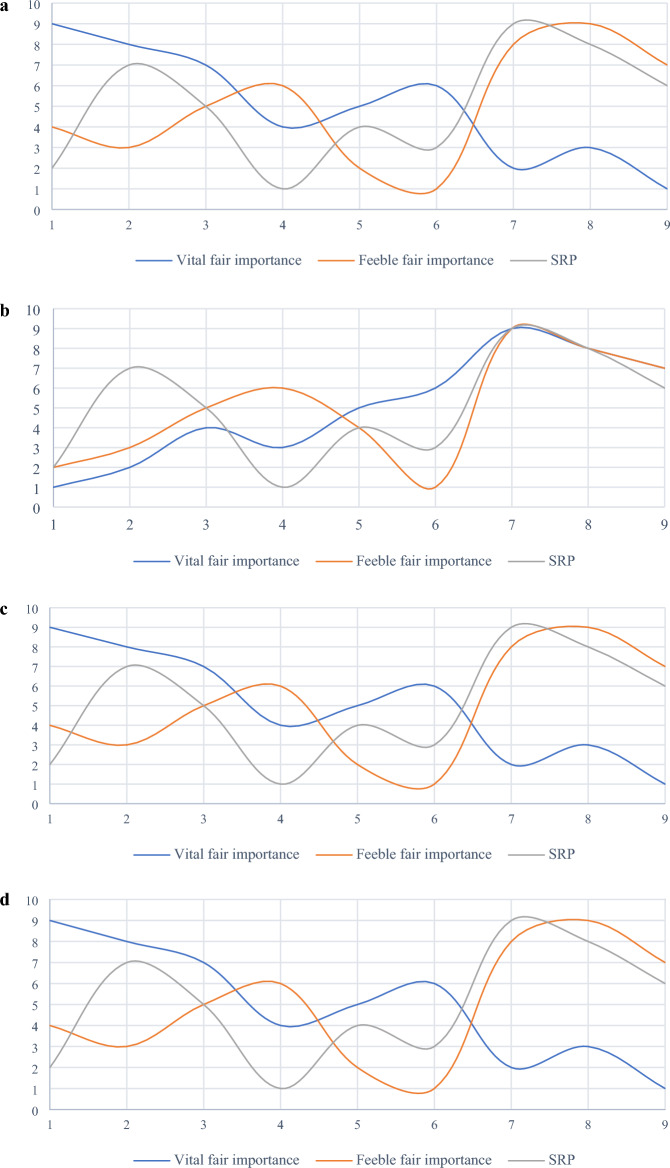

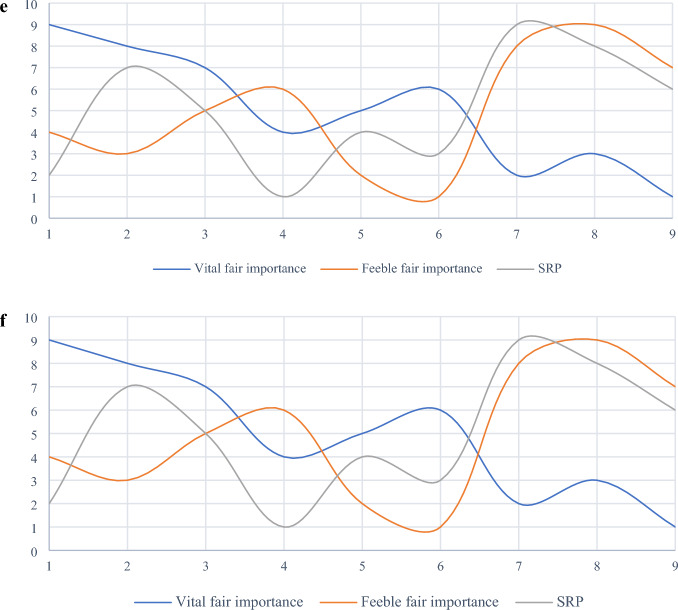
(**a**) Changes in ranking, affected by the vital and feeble fair importance values, associated with weight of ${c}_{1}$. (**b**) Changes in ranking, affected by the vital and feeble fair importance values, associated with weight of ${c}_{2}$ criterion. (**c**) Changes in ranking, affected by the vital and feeble fair importance values, associated with weight of ${c}_{3}$ criterion. (**d**) Changes in ranking, affected by the vital and feeble fair importance values, associated with weight of ${c}_{4}$ criterion. (**e**) Changes in ranking, affected by the vital and feeble fair importance values, associated with weight of ${c}_{5}$ criterion. (**f**)Changes in ranking, affected by the vital and feeble fair importance values, associated with weight of ${c}_{6}$ criterion.

**Figure 13:**
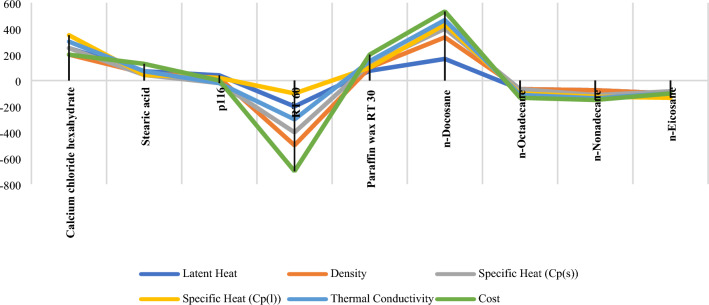
Dependency of each material on each criterion.

Figure 12 depict the changes in each material's rank caused by applying each criterion's corresponding fair vital importance and fair feeble importance compared to the original ranking associated with the criteria's real importance. Compared to the changes in overall ranks affected by fair vital importance in overall rankings, almost no changes were detected in the rankings affected by the feeble fair criteria, which indicates that the SRP becomes more sensitive to weight changes by increasing the number of criteria. Figure 13, developed using Eq. (33), displays the dependency of the ranking generated by SRP on each criterion.

By applying Eq. (35), it has been found that the overall dependency of SRP method is 0.513, which indicates a value of Ω ≤ 0.50, slightly exceeding the limit established for an MCDM method's reliability. This indicates that SRP method is reliable in analyzing problems with six criteria. Since SRP method employs the ranks of each alternative in each criterion, and the criteria weight plays a critical role in determining the generated rankings, reliability of the method improves with increasing criteria, making it an effective tool for solving complex MCDM problems.

### The comparative rankings analysis—Similarity measures

In this section, the obtained ranks from different MCDM methods have been evaluated through six similarity measures, including Manhattan distance and total similarity Eqs. (36) and (37), Canberra distance and total similarity Eqs. (38) and (39), Chi-square distance and total similarity Eqs. (40) and (41), and Squared Euclidean distance and total similarity Eqs. (42) and (43), in order to conclude the similarities between SRP and other MCDM methods Eq. (44). In the equations, ${R}_{x}$, ${R}_{y}$, ${\mathbb{R}}_{i}^{x}$, and ${\mathbb{R}}_{i}^{y}$ represent the output of $x$th MCDM method, $y$th MCDM method, $i$th alternative’s rank in $x$th MCDM method, and the same alternative’s rank in $y$th MCDM method, respectively. ${\mathrm{\rm T}d}_{\left({R}_{x},{R}_{V}\right)}$ denote the total similarity between $x$th MCDM method and $y$*th* MCDM method, in which $\aleph$ signifies the total number of MCDM methods that have been used for similarity evaluation.

37 $${d}_{1}\left({R}_{x},{R}_{y}\right)=\sum_{i=1}^{m}\left|{\mathbb{R}}_{i}^{x}-{\mathbb{R}}_{i}^{y}\right|, i=\left\{1,..,m\right\}, {R}_{x}=\left\{{\mathbb{R}}_{1}^{x},...,{\mathbb{R}}_{m}^{x}\right\}, {R}_{y}=\left\{{\mathbb{R}}_{1}^{y},...,{\mathbb{R}}_{m}^{y}\right\},$$

38 $${d}_{1}^{^{\prime}}\left({R}_{x},{R}_{V}\right)= \langle \sum_{i=1}^{m}\left|{\mathbb{R}}_{i}^{x}-{\mathbb{R}}_{i}^{y}\right|{\langle \sum_{V=1}^{g}\sum_{i=1}^{m}\left|{\mathbb{R}}_{i}^{x}-{\mathbb{R}}_{i}^{y}\right|\rangle }^{-1}\rangle \times 100, y\in V, V=\left\{1,\dots , g\right\},$$

39 $${d}_{2}\left({R}_{x},{R}_{y}\right)=\sum_{i=1}^{m}\frac{\left|{\mathbb{R}}_{i}^{x}-{\mathbb{R}}_{i}^{y}\right|}{\left|{\mathbb{R}}_{i}^{x}+{\mathbb{R}}_{i}^{y}\right|},$$

40 $${d}_{2}^{^{\prime}}\left({R}_{x},{R}_{V}\right)=\langle \sum_{i=1}^{m}\frac{\left|{\mathbb{R}}_{i}^{x}-{\mathbb{R}}_{i}^{y}\right|}{\left|{\mathbb{R}}_{i}^{x}+{\mathbb{R}}_{i}^{y}\right|}{\langle \sum_{V=1}^{g}\sum_{i=1}^{m}\frac{\left|{\mathbb{R}}_{i}^{x}-{\mathbb{R}}_{i}^{y}\right|}{\left|{\mathbb{R}}_{i}^{x}+{\mathbb{R}}_{i}^{y}\right|}\rangle }^{-1}\rangle \times 100,$$

41 $${d}_{3}\left({R}_{x},{R}_{y}\right)=\sum_{i=1}^{m}\frac{{\left({\mathbb{R}}_{i}^{x}-{\mathbb{R}}_{i}^{y}\right)}^{2}}{{\mathbb{R}}_{i}^{x}+{\mathbb{R}}_{i}^{y}}, $$

42 $${d}_{3}^{^{\prime}}\left({R}_{x},{R}_{V}\right)=\langle \sum_{i=1}^{m}\frac{{\left({\mathbb{R}}_{i}^{x}-{\mathbb{R}}_{i}^{y}\right)}^{2}}{{\mathbb{R}}_{i}^{x}+{\mathbb{R}}_{i}^{y}}{\langle \sum_{V=1}^{g}\sum_{i=1}^{m}\frac{{\left({\mathbb{R}}_{i}^{x}-{\mathbb{R}}_{i}^{y}\right)}^{2}}{{\mathbb{R}}_{i}^{x}+{\mathbb{R}}_{i}^{y}}\rangle }^{-1}\rangle \times 100,$$

43 $${d}_{4}\left({R}_{x},{R}_{y}\right)=\sum_{i=1}^{m}{\left({\mathbb{R}}_{i}^{x}-{\mathbb{R}}_{i}^{y}\right)}^{2}, $$

44 $${d}_{4}^{^{\prime}}\left({R}_{x},{R}_{V}\right)=\langle \sum_{i=1}^{m}{\left({\mathbb{R}}_{i}^{x}-{\mathbb{R}}_{i}^{y}\right)}^{2}{\langle \sum_{V=1}^{g}\sum_{i=1}^{m}{\left({\mathbb{R}}_{i}^{x}-{\mathbb{R}}_{i}^{y}\right)}^{2}\rangle }^{-1}\rangle \times 100,$$

45 $${\mathrm{\rm T}d}_{\left({R}_{x},{R}_{V}\right)}=\langle \frac{{d}_{1}^{^{\prime}}\left({R}_{x},{R}_{V}\right)}{\sum {d}_{1}^{^{\prime}}\left({R}_{x},{R}_{V}\right)}+\frac{{d}_{2}^{^{\prime}}\left({R}_{x},{R}_{V}\right)}{\sum {d}_{2}^{^{\prime}}\left({R}_{x},{R}_{V}\right)}+\frac{{d}_{3}^{^{\prime}}\left({R}_{x},{R}_{V}\right)}{\sum {d}_{3}^{^{\prime}}\left({R}_{x},{R}_{V}\right)}+\frac{{d}_{4}^{^{\prime}}\left({R}_{x},{R}_{V}\right)}{\sum {d}_{4}^{^{\prime}}\left({R}_{x},{R}_{V}\right)}\rangle {\aleph }^{-1},$$

#### Manhattan distance

**Figure 14:**
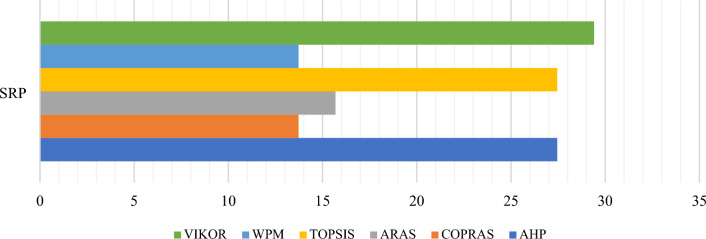
Comparative analysis of the similarity between SRP and other MCDM methods using Manhattan distance.

Using Manhattan distance to evaluate the similarities between SRP and VIKOR, WPM, TOPSIS, ARAS, COPRAS, and AHP showed that VIKOR has the most resemblance in evaluating materials with SRP. The results of using Manhattan distance are illustrated in Fig. 14.

#### Canberra distance

**Figure 15:**
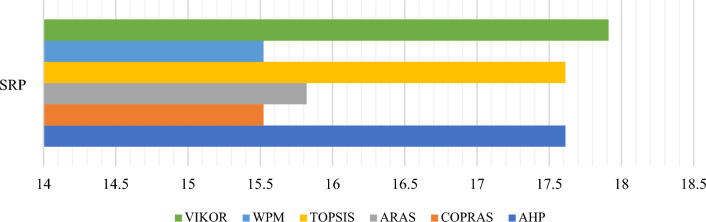
Comparative analysis of the similarity between SRP and other MCDM methods using Canberra distance.

Similar to the Manhattan distance, the Canberra distance puts VIKOR most resembling SRP. Applying the Canberra distance puts AHP higher than TOPSIS as the second most resemblance method to the SRP in solving the material selection problem. In contrast, the Manhattan distance considers TOPSIS the second most resemblance method to the SRP with a slightly higher score (see Table 29). The results of the Canberra distance application are pictured in Fig. 15.

**Table 29:** Results of each similarity measure method.

Distance	AHP	COPRAS	ARAS	TOPSIS	WPM	VIKOR
Manhattan distance	27.4509	13.7255	15.6860	27.4510	13.725	29.4117
Canberra distance	17.6120	15.5224	15.8209	17.6119	15.5223	17.9104
Chi-square distance	21.8610	11.2600	11.2470	21.2340	9.0500	25.3480
Hamming distance	22.3048	10.0371	10.7806	23.7918	7.8066	25.2788

#### Chi-square distance

**Figure 16:**
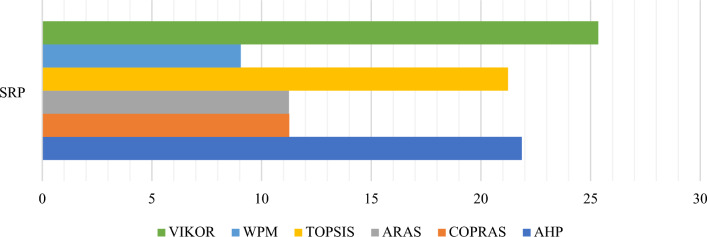
Comparative analysis of the similarity between SRP and other MCDM methods using Chi-square distance.

The Chi-square distance analysis reveals that AHP is the second most similar MCDM method to SRP, while WPM is the least similar. In comparison to Manhattan and Canberra distances, where COPRAS and WPM have almost equal scores, Chi-square distance gives WPM the lowest similarity score and the farthest distance from the COPRAS method. The results of this comparison are shown in Fig. 16.

#### Squared Euclidean distance

**Figure 17:**
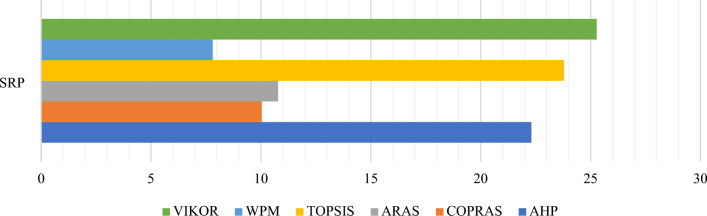
Comparative analysis of the similarity between SRP and other MCDM methods using Squared Euclidean distance.

Except for VIKOR which has been placed by Squared Euclidean distance as the most similar method to SRP dominantly, TOPSIS is the second MCDM method that shows similarity to SRP in ranking the materials. The results of Squared Euclidean distance application are portrayed in Fig. 17.

#### Total similarity

**Figure 18:**
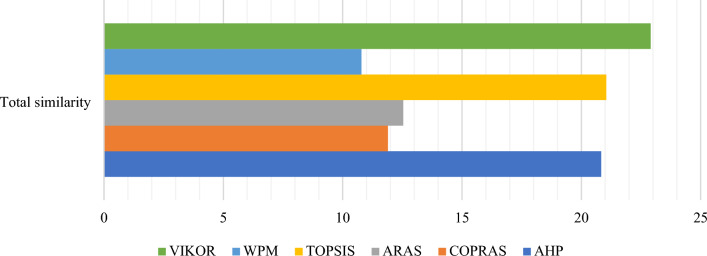
Total similarity of each MCDM method to SRP.

"Total similarity" as computed using Eq. (44), has generated almost identical rankings of similarity, where VIKOR, TOPSIS, and AHP, with a considerable distance, are the most similar methods to the SRP method, respectively. On the other hand, ARAS, COPRAS, and WPM showed the most dissimilarity compared to SRP. The results are demonstrated in Table 29 and Fig. 18.

### The rank reversal phenomenon: A comparison between SRP and other rank reversal free MCDM methods

The rank reversal phenomenon refers to the occurrence of changes in the relative rankings of alternatives when additional alternatives are introduced or existing alternatives are removed from a set being evaluated in MCDM environment^99^. This phenomenon can lead to inconsistent decisions and can make it challenging to compare and evaluate alternatives across different decision problems. Several MCDM methods have been proven to be rank reversal-free, including Characteristic Objects Method (COMET) proposed by Piegat & Sałabun^100^ and Sałabun^101^, Stable Preference Ordering Towards Ideal Solution (SPOTIS) developed by Dezert^102^, Ranking of Alternatives through Functional mapping of criterion sub-intervals into a Single Interval (RAFSI) proposed by Žižović et al.^103^, and the Sequential Interactive Model of Urban Systems (SIMUS) developed by Munier^104^. Among these methods, COMET method is the first MCDM method that is completely immune to the rank reversal paradox. COMET method considers the correlations between the criteria, and provides ranking by considering the characteristic objects and fuzzy rules^101^. COMET method has proven to be robust and effective in avoiding the rank reversal paradox in various applications, such as those described in Wątróbski et al.^105^^,^ Shekhovtsov et al.^106^^,^ Faizi et al.^107^^,^ and Palczewski & Sałabun^108^. To test the rank reversal paradox of SRP, three examples are provided including a material selection case and two other numerical examples.

#### The material selection case

**Figure 19:**
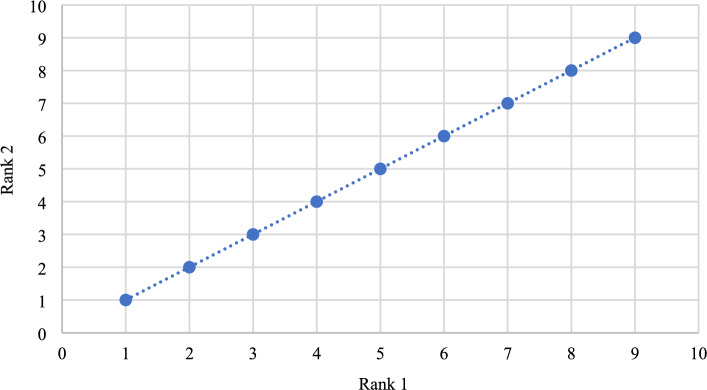
The correlation between two ranks to assess the rank reversal incident.

A new alternative is added to the original material selection decision matrix (Table 5), in which ${A}_{4}^{*}$ stands for the new alternative and its performance is equal to ${A}_{4}$. The new decision matrix and the corresponding ranks given by SRP are shown in Table 30. In order to demonstrate the correlation between the original ranking and the obtained ranking, Fig. 19 is presented, where a correlation of 1 is indicated, suggesting that SRP is a rank reversal free MCDM method.

**Table 30:** New ranking given by SRP after adding the new alternative.

PCM	${c}_{1}$	${c}_{2}$	${c}_{3}$	${c}_{4}$	${c}_{5}$	${c}_{6}$	${SR}_{i}$	${\mathfrak{R}}_{i}$	Original rank	New rank
A1	169.98	1560	1.46	2.13	1.09	0.255	4.269	4.731	2	2
A2	186.5	903	2.83	2.38	0.18	0.745	5.012	3.988	7	7
A3	190	830	2.1	2.1	0.21	0.335	4.68	4.32	5	5
A4	214.4	850	0.9	0.9	0.2	0.255	4.084	4.916	1	1
A5	206	789	1.8	2.4	0.18	0.335	4.674	4.326	4	4
A6	194.6	785	1.93	2.38	0.22	0.335	4.321	4.679	3	3
A7	245	773.22	0.3767	2.267	0.14	0.335	5.545	3.455	9	9
A8	222	775.8	1.7189	1.921	0.142	0.665	5.339	3.661	8	8
A9	247	776.33	0.7467	2.377	0.138	0.336	4.981	4.019	6	6
A4*	214.4	850	0.9	0.9	0.2	0.255

### The first numerical example

**Figure 20:**
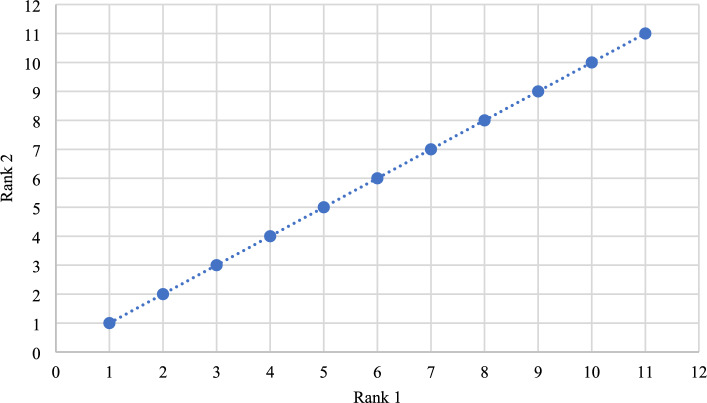
Correlation between two obtained rankings for the first numerical example.

A numerical example is presented in Table 31, comprising of eleven alternatives and fifteen criteria. All the criteria are beneficial, having equal weights. The ranking of alternatives is shown in Table 32, where A6 is assigned the first rank. In the case of adding a new alternative with the same performance as A6, no changes have been observed in the ranks, and the correlation between the two rankings is one, as shown in Table 33 and Fig. 20.

**Table 31:** Decision matrix of the first numerical example.

Weight	0.067	0.067	0.067	0.067	0.067	0.067	0.067	0.067	0.067	0.067	0.067	0.067	0.067	0.067	0.067
Alternative	C1	C2	C3	C4	C5	C6	C7	C8	C9	C10	C11	C12	C13	C14	C15
A1	0.087	0.046	3.252	0.737	0.092	1.318	2.912	0.074	0.054	0.067	1.505	1.599	1.219	0.056	0.031
A2	0.002	0.094	0.732	3.564	2.545	2.059	1.860	0.056	0.070	0.085	1.265	0.116	0.783	0.080	0.005
A3	0.047	0.052	2.485	1.599	3.900	0.267	1.477	0.089	0.067	0.096	0.898	1.037	0.821	0.028	0.088
A4	0.093	0.011	1.336	1.788	1.804	3.879	2.650	0.001	0.041	0.050	0.023	0.976	1.057	0.002	0.010
A5	0.058	0.084	2.777	3.025	0.814	1.256	1.136	0.034	0.054	0.078	0.606	1.650	0.805	0.013	0.032
A6	0.017	0.032	3.353	2.240	3.840	2.005	2.250	0.030	0.099	0.080	0.578	1.660	0.669	0.030	0.083
A7	0.075	0.050	0.467	0.681	0.960	1.986	1.998	0.002	0.096	0.084	0.660	0.800	1.673	0.089	0.002
A8	0.011	0.051	2.966	0.639	0.959	3.116	0.424	0.097	0.089	0.089	0.438	0.944	0.544	0.041	0.080
A9	0.080	0.096	3.819	3.948	2.994	3.610	1.725	0.001	0.017	0.030	0.221	1.516	0.755	0.064	0.017
A10	0.002	0.035	2.546	0.787	3.038	0.552	1.946	0.002	0.007	0.050	0.068	0.397	0.127	0.029	0.002
A11	0.048	0.011	1.675	3.243	0.306	1.696	1.732	0.081	0.068	0.059	0.334	0.342	0.348	0.038	0.031

**Table 32:** The different alternatives’ rankings to test the rank reversal paradox.

Alternative	${SR}_{i}$	${\mathfrak{R}}_{i}$	${{SR}_{i}}_{2}$	${{\mathfrak{R}}_{i}}_{2}$	Original rank	New rank
A1	5.0000	6.0000	5.5333	6.4667	2	2
A2	5.4000	5.6000	5.8667	6.1333	4	4
A3	5.1333	5.8666	5.6000	6.4000	3	3
A4	6.9333	4.0666	7.6667	4.3333	9	9
A5	6.0666	4.9333	6.6667	5.3333	8	8
A6	4.7333	6.2666	4.7333	7.2667	1	1
A7	5.7333	5.2666	6.3333	5.6667	6	6
A8	5.8666	5.1333	6.5333	5.4667	7	7
A9	5.4666	5.5333	6.0000	6.0000	5	5
A10	8.600	2.40000	9.5333	2.4667	11	11
A11	7.0666	3.9333	7.8000	4.2000	10	10

**Table 33:** Two obtained rankings from SRP application for the second numerical example.

Weight	0.25	0.25	0.25	0.25						
Alternative	C1	C2	C3	C4	${SR}_{i}$	${\mathfrak{R}}_{i}$	${{SR}_{i}}_{2}$	${{\mathfrak{R}}_{i}}_{2}$	Original rank	New rank
A1	912.4845	0.3018	206.4309	0.1221	12.75	7.25	13.5	7.5	14	14
A2	517.0006	0.1986	616.1050	0.0945	13.5	6.5	14.5	6.5	16	16
A3	19.61708	0.6361	348.3409	0.1985	14.5	5.5	15.5	5.5	17	17
A4	1069.093	0.9259	918.6073	0.0005	8.25	11.75	9	12	5	5
A5	151.0204	0.0969	376.8284	0.2074	15.25	4.75	16.25	4.75	19	19
A6	1022.647	0.2007	430.3449	0.3781	9	11	9.75	11.25	6	6
A7	944.3263	1.2419	443.6463	0.2520	6.75	13.25	7.5	13.5	4	4
A8	974.168	0.2407	978.1523	0.0137	10.25	9.75	10.75	10.25	9	9
A9	504.9541	1.2165	176.6877	0.3398	10	10	11	10	8	8
A10	549.4893	0.2430	645.7940	0.2352	10.5	9.5	11.5	9.5	10	10
A11	319.5062	1.1863	57.60621	0.3537	11	9	12	9	12	12
A12	86.49311	0.6288	258.042	0.0811	15.5	4.5	16.5	4.5	20	20
A13	991.6532	0.6623	962.0012	0.3963	4	16	4.25	16.75	2	2
A14	279.4132	1.0169	688.305	0.0302	11.5	8.5	12.5	8.5	13	13
A15	787.9316	2.1867	926.4695	0.3922	3.75	16.25	3.75	17.25	1	1
A16	866.5065	1.0388	1044.6860	0.2068	6	14	6.5	14.5	3	3
A17	580.661	0.3445	430.0089	0.0343	13.25	6.75	14.25	6.75	15	15
A18	407.2574	0.5822	659.9484	0.3830	9	11	10	11	6	6
A19	460.7242	0.0873	491.7818	0.1171	14.5	5.5	15.5	5.5	17	17
A20	45.74425	0.7103	643.1528	0.2860	10.75	9.25	11.75	9.25	11	11

### Second numerical example

**Figure 21:**
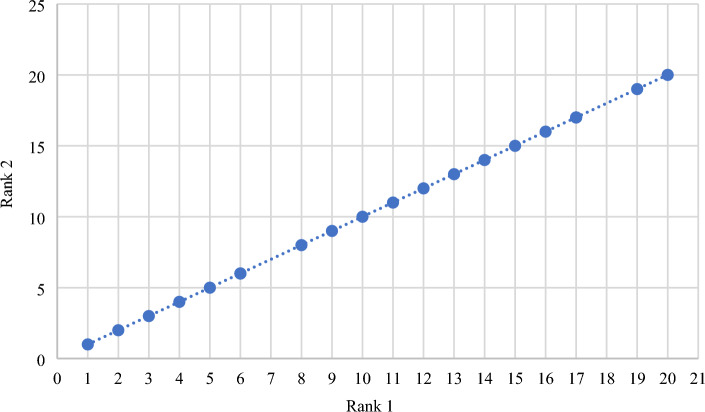
The correlation between two obtained rankings from the second numerical example.

An additional example is presented to represent a more complex MCDM problem with a larger number of alternatives and fewer criteria. This example includes twenty alternatives and four criteria, each with equal weights, as shown in Table 33. To illustrate the robustness of SRP, a new alternative with the same performance as the original top-ranked alternative (A15) is added, resulting in no changes in the ranking and a correlation coefficient of one, as shown in Fig. 21. This provides further evidence that SRP is a rank reversal-free MCDM method.

The above examples revealed that the SRP method is utterly immune to the rank reversal paradox. As mentioned earlier, SPOTIS, COMET, and SIMUS are also rank reversal-free MCDM methods. The complexity of any MCDM method can lead to less transparency and uncertainty in its outcomes, especially in complex systems with many interacting parts and variables. Increased complexity of an MCDM method also hinders the ability to track and identify errors in the algorithmic output. Complex systems, such as those with numerous pairwise comparisons or different normalization approaches, are more likely to produce uncertain outcomes due to the complex interplay between variables. One of the major benefits of SRP in this context is its simplicity which helps to avoid those issues and provides reliable results when the problem becomes more complex.

## Conclusions and future research

Material selection problems are complex MCDM problems that comprise many options and criteria. To solve material selection problems, a new simple MCDM method called SRP is proposed in this paper. The new method functions based on the different ranks of the material in each criterion to generate reliable outputs in solving MCDM problems. One of the sources of anomalies in comparison processes of the generated results of MCDM methods is the different normalization strategies employed by different MCDM methods. To enhance the reliability of results in complex MCDM problems, SRP method avoids the normalization process of the decision matrix and operates solely with the ranks of alternatives, enabling a comparison of scores with the same unit. In a material selection problem that involved selecting a suitable phase change material to store solar energy, SRP method was employed. However, since SRP relies heavily on the criteria weights in generating rankings, it is essential to have a dependable method for determining these weights. In this study, VIMM method was utilized to extract the criteria weights through a decision-making process involving seven experts. By utilizing the newly proposed method to validate MCDM methods based on the analysis of their dependency on criteria weights, it was found that the SRP method is sensitive to changes in the criteria weights. Even slight changes in the weights can significantly affect the ranks of materials obtained by the SRP method. We also compared the results obtained from the application of other MCDM methods and found significant differences between the generated results. In this paper, CDI has been introduced as a new statistical measure to validate the results. CDI is used to interpret the results of MCDM methods in four categories, namely pragmatic compromise, rational compromise, fair compromise, and rotten compromise. The most reliable results were placed under the pragmatic compromise category. On the other hand, undependable results were interpreted according to the fair compromise and the rotten compromise situations, and their comparison needed to be executed in the real world to determine which method was better, while the mathematical proof was enough in the first category. The comparison results were put under the fair compromise category by the results of *CDI*. The new measure will decrease the cost of wrong material selection. The obtained ranking from SRP is also compared with other MCDM methods using Zakeri-Konstantas Performance Correlation Coefficient, which showed that the new method is more similar to the COPRAS than AHP and ARAS. Four different similarity measures are also applied to evaluate the similarity between other MCDM methods to SRP which has some salient advantages to make it an ideal MCDM method for solving complex problems. Overall, with the employed comparison processes, it is concluded that:

1. SRP produces more reliable products since it does not execute the normalization process.
2. SRP is a reliable MCDM method since the analytical processes showed that it is sensitive to changes in the criteria weights.
3. SRP is designed to generate ranking in a less complex analysis which correlates to less uncertainty in the final results.
4. The algorithm of SRP is easy to reverse track by decision-makers to identify possible errors.
5. The reliability of SRP increases by increasing the number of criteria, making it ideal for solving complex decision-making problems involving a large number of criteria and alternatives.
6. According to the results obtained from evaluation of the similarities between other MCDM methods and SRP, it is observed that SRP behaves similar to distance-based methods, e.g. VIKOR and TOPSIS, and also shows a resemblance to AHP in some results.

The results also revealed a limitation of SRP. The obtained results are not potentially reliable for those MCDM problems where the number of criteria is less than six, which makes it an ideal method for solving complex MCDM problems involving higher number of criteria. Future research would be interesting in assessing the applicability and validation of the new method. Application and validation of the results of SRP for solving other material selection problems is the second suggestion for future research. Validation of the interpretation of *CDI* by simulation is a very interesting future study work. Other intriguing ideas for future research include comparing the interpretation with other statistical techniques and CDI to validate the outcomes. Integrating criteria weights from different weighting methods, including both subjective and objective methods, with dependency analysis to evaluate and validate the result is another exciting suggestion for future research. This paper used VIMM: the first scenario as a weighting method because of its reliability. It is also suggested to use AHP and BWM in conjunction with SRP to comparing the results as future research scope. Development of extensions of SRP for resolving different types of MCDM problems with multiple-layer criteria is the concluding suggestion for further research. 

## Data Availability

The datasets used and/or analysed during the current study available from the corresponding author on reasonable request.
